# Synergistic Effects of SGLT2 Inhibitors and GLP-1R Agonists on Inflammation-Associated Oxidative Stress in Atrial Fibrillation

**DOI:** 10.1016/j.jacbts.2026.101587

**Published:** 2026-06-06

**Authors:** Walaa Fakih, Ali Mroueh, Shinnosuke Kikuchi, Halim Marzak, Said Amissi, Dal-Seong Gong, Sophie Kerth, Michel Kindo, Arnaud Mommerot, Michael Paul Pieper, Patrick Ohlmann, Olivier Morel, Valérie Schini-Kerth, Laurence Jesel

**Affiliations:** a University of Strasbourg, UR 3074, Translational Cardiovascular Medicine, FMTS, Biomedicine Research Center of Strasbourg, Strasbourg, France; b Cardiology Department, Strasbourg University Hospital, Strasbourg, France; c Experimental and Molecular Pediatric Cardiology, TUM University Hospital, German Heart Center Munich, Technical University Munich, Munich, Germany; d Boehringer Ingelheim Pharma, Global Cardio-Metabolic Diseases, Biberach an der Riss, Germany

**Keywords:** atrial fibrosis, atrial remodeling, glucagon-like peptide-1 receptor, inflammation, oxidative stress, sodium-glucose cotransporter 2

## Abstract

Atrial fibrillation (AF) is linked to atrial inflammation and adverse remodeling. The authors analyzed 80 right atrial appendages from cardiac surgery patients, including 18 with AF history, to examine the relationship between inflammation and sodium-glucose cotransporter 2 (SGLT2) and glucagon-like peptide 1 receptor (GLP-1R) expression and function. Inflamed right atrial appendages showed increased oxidative stress, fibrosis, and SGLT2 and GLP-1R expression, which were more pronounced in patients with AF. SGLT2 inhibitors and GLP-1R agonists reduced reactive oxygen species levels, with combination therapy being most effective. These findings suggest combined SGLT2 inhibitor and GLP-1R agonist treatment could be a potential approach to prevent AF-related atrial remodeling by reducing oxidative stress.

## Highlights

• RAA from AF patients show higher inflammation and oxidative stress vs non-AF
• RAA inflammation correlates with SGLT2 and GLP-1R expression.
• SGLT2 and GLP-1R modulation exert synergistic antioxidant effects in RAA
• Combined SGLT2i/GLP-1Ra therapy represents a promising strategy against AF remodeling

## Visual Abstract

**Figure:**
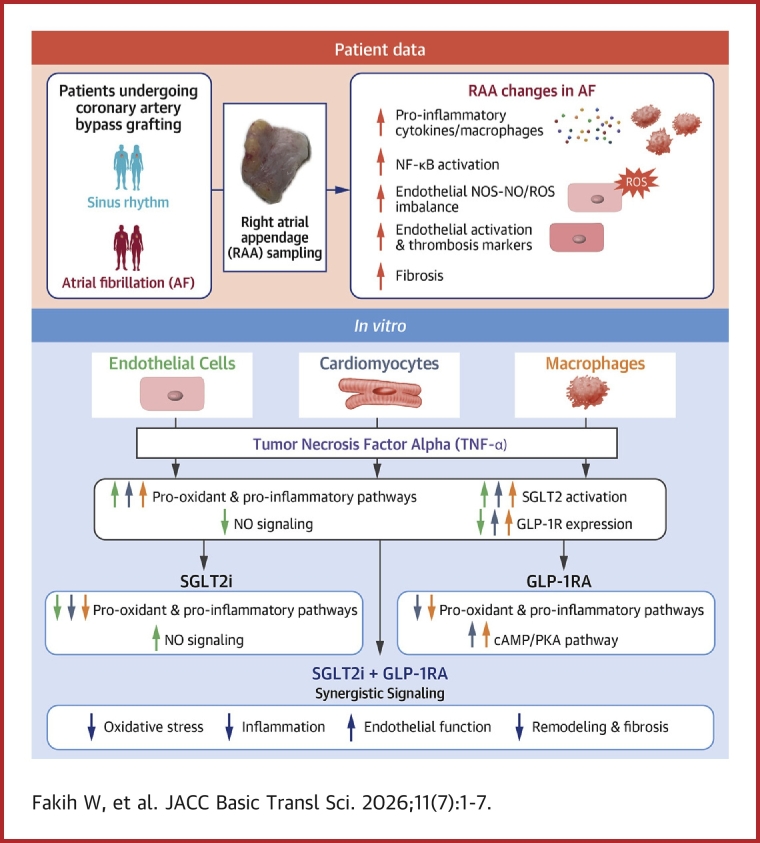

Atrial fibrillation (AF) is the most common cardiac arrhythmia. It is a major cause of stroke and is strongly associated with heart failure (HF), particularly HF with preserved ejection fraction. Progressive electrical and structural remodeling of the atria underlie the concept of atrial cardiomyopathy, which often precedes the development of AF.^1^ A major component of atrial cardiomyopathy is chronic inflammation, which creates a microenvironment that favors profibrotic remodeling and can ultimately lead to AF.^2^ Currently, there are limited therapeutics that directly address atrial inflammation and remodeling in AF pathogenesis.

Sodium-glucose cotransporter 2 inhibitors (SGLT2i), a class of diabetes drugs, have demonstrated marked beneficial effects in reducing cardiovascular mortality and HF-related hospitalizations in both diabetic and nondiabetic populations.^3^ Although the mechanisms by which SGLT2i improve cardiovascular outcomes are not fully understood, they have been proposed to regulate oxidative stress and inflammation in the heart.^4^ Indeed, proinflammatory cytokines can up-regulate sodium-glucose cotransporter 2 (SGLT2) expression through reactive oxygen species (ROS)–dependent mechanisms and promote profibrotic tissue remodeling.^5^ Interestingly, SGLT2i have also been associated with reduced AF incidence^6^ and lower AF recurrence after ablation.^7^ However, the mechanisms by which SGLT2i attenuate AF risk are largely unclear.

More recently, glucagon-like peptide 1 receptor agonists (GLP-1Ra), another class of diabetes medications, have been shown to improve outcomes in obesity-related HF with preserved ejection fraction, which is strongly linked to AF.^8^ Interestingly, GLP-1Ra improve adverse cardiac remodeling, including reducing left atrial (LA) volume, in patients with obesity-related HF with preserved ejection fraction.^9^ Similar to SGLT2i, GLP-1Ra also demonstrate anti-inflammatory and antioxidant effects, both systemically and in the heart.^10^^,^^11^

Although emerging clinical data now suggests that both SGLT2i and GLP-1Ra may also have beneficial effects in AF, the mechanisms underlying these effects are not clearly understood. Using right atrial appendage (RAA) samples from patients undergoing cardiac surgery, the aim of this study was to deepen our understanding of SGLT2 and glucagon-like peptide 1 receptor (GLP-1R) biology in atrial inflammation and remodeling and test the hypothesis that SGLT2i and GLP-1Ra may have synergistic effects on adverse atrial remodeling in AF.

## Methods

Additional detailed materials and methods are available in the Supplemental Appendix.

### Human RAA sample preparation

RAA samples were obtained from 80 patients undergoing coronary artery bypass grafting or aortic valve replacement at the University Hospital of Strasbourg in France. The study complied with the Declaration of Helsinki and was approved by the local Ethics Committee (EK2044, March 4, 2013). All patients provided written informed consent prior to enrollment. Patients with relevant comorbidities, including malignancies or chronic inflammatory diseases, were excluded. Preoperative evaluation included medical history, transthoracic echocardiography, European System for Cardiac Operative Risk Evaluation score assessment, 12-lead electrocardiography, and routine hematologic and biochemical testing. All patients were in sinus rhythm (SR) at the time of surgery. RAA samples were immediately snap-frozen in liquid nitrogen or embedded in Tissue-Tek optimal cutting temperature compound (Sakura Finetek).

### Statistical analysis

Continuous data for RAA samples are expressed as mean ± SEM. Statistical analyses were performed using Prism version 9.0 (GraphPad Software). Groups were compared using an unpaired Student’s *t*-test, multiple Student’s *t*-tests with Benjamini-Hochberg correction for false discovery rate, or 1- or 2-way analysis of variance followed by Tukey’s post hoc test for multiple pairwise comparisons as appropriate. Data normality was assessed using the Kolmogorov-Smirnov test prior to statistical testing. Correlations were analyzed using Pearson’s test for normally distributed variables or Spearman’s test otherwise, and results are reported as the correlation coefficient (*r*) and coefficient of determination (*r*^2^). Clinical data are expressed as median (Q1-Q3) or count (percentage) unless otherwise specified and analyzed using JMP Pro 16 (SAS Institute).

To reduce confounding from baseline differences, inverse probability of treatment weighting (IPTW) was applied using propensity scores derived from a logistic regression model including age, sex, body mass index, hypertension, diabetes, left ventricular ejection fraction <60%, and stroke. Covariate balance before and after IPTW adjustment was evaluated using standardized mean differences. A standardized mean difference <0.25 was considered acceptable in this study.^12^ Comparisons of marker levels between patients in SR and those with AF were performed in the IPTW-weighted population.

Associations between clinical variables and molecular markers were evaluated using univariable and multivariable analyses in the unweighted cohort. Variables with *P* < 0.05 in univariable analysis were included in multivariable models, and confounding parameters were excluded. Comparisons between patients in the SR group and those with histories of AF were performed using the chi-square test for categorical variables and the Mann-Whitney *U* test for continuous variables reported as median (Q1-Q3). Sample sizes are indicated in each figure legend, and a *P* value <0.05 was considered to indicate statistical significance.

## Results

### Patient population studied

Demographic and clinical characteristics are summarized in Table 1. In total, RAA samples were collected from 80 patients undergoing cardiac surgery, of whom 18 had histories of AF (77.8% paroxysmal, 22.2% persistent). Patients with AF were older (median age 76 years; Q1-Q3: 62-78 years), had higher CHA_2_DS_2_-VASc scores, and exhibited larger LA volume index and area compared with patients with no history of AF (Table 1). Left ventricular ejection fraction was notably lower in patients with AF (median 60.0%; Q1-Q3: 58.0%-63.5%). Regarding medication use, there was a higher prevalence of angiotensin II type 1 receptor antagonist (44.4% vs 21.0%), direct oral anticoagulant agent (55.6% vs 6.5%), and vitamin K antagonist (22.2% vs 4.8%) (Table 1) use in patients with AF compared with patients without AF. For simplicity, from here on, patients without histories of AF are subsequently referred to as SR patients.

**Table 1:** Demographic Characteristics of the Study Participants

	Whole Cohort (N = 80)	SR (n = 62)	AF (n = 18)	*P* Value
Age, y	66 (56-75)	65 (55-71)	76 (61.8-78.0)	**0.003**
Age ≥75 y	19 (23.8)	9 (14.5)	10 (55.6)	**0.001**
Male	64 (80.0)	48 (77.4)	16 (88.9)	0.28
Hypertension	45 (56.3)	32 (51.6)	13 (72.2)	0.12
Dyslipidemia	39 (48.8)	29 (46.8)	10 (55.6)	0.51
Diabetes	13 (16.2)	10 (16.1)	3 (16.7)	0.96
Smoking	22 (27.5)	20 (32.3)	2 (11.1)	0.077
Body mass index, kg/m^2^	26.6 (23.3- 29.2)	26.8 (23.3-29.9)	25.0 (22.8-28.0)	0.19
History of stroke	1 (1.3)	0 (0.0)	1 (5.6)	0.062
Vascular pathology	19 (23.8)	13 (20.8)	6 (33.3)	0.28
Paroxysmal AF	14 (17.5)	0 (0.0)	14 (77.8)	**0.001**
Persistent AF	4 (5.0)	0 (0.0)	4 (22.2)	**0.001**
CHA_2_DS_2_-VASc score				
0 or 1	53 (66.3)	46 (74.2)	7 (38.9)	**0.005**
2 or 3	21 (26.3)	14 (22.6)	8 (44.4)	0.067
≥4	5 (6.3)	2 (3.2)	3 (16.7)	**0.038**
LA volume index, mL/m^2^	37 (26.2-51.0)	34 (25.0-45.5)	49 (33.0-61.0)	**0.005**
LA area, cm^2^	22.6 (18.0-26.2)	21.0 (17.0-26.0)	25.0 (20.0-31.0)	**0.023**
Aortic stenosis	21 (26.3)	16 (25.8)	5 (27.8)	0.86
LVEF, %	63.5 (60.0-68.0)	65.0 (61.0-68.6)	60.0 (58.0-63.5)	**0.035**
LVEF <60%	17 (21.3)	11 (17.7)	6 (33.3)	0.15
Creatinine clearance, mL/min	74.6 (65.1-89.5)	74.0 (65.0-88.0)	76.9 (66.0-96.0)	0.42
eGFR <50 mL/min/1.73 m^2^	3 (3.8)	2 (3.2)	1 (5.6)	0.65
Statins	36 (43.8)	25 (40.3)	11 (61.1)	0.12
Aldosterone antagonists	3 (3.8)	1 (1.6)	2 (11.1)	0.062
ACE inhibitors	17 (21.3)	13 (21.0)	4 (22.2)	0.91
AT1R antagonists	21 (26.3)	13 (21)	8 (44.4)	**0.046**
DOACs	14 (17.5)	4 (6.5)	10 (55.6)	**0.001**
VKAs	7 (8.8)	3 (4.8)	4 (22.2)	**0.022**

Values are median (Q1-Q3) or n (%). *P* values in boldface type denote statistical significance.

ACE = angiotensin-converting enzyme; AF = atrial fibrillation; AT1R = angiotensin type 1 receptor; DOAC = direct oral anticoagulant agent; eGFR = estimated glomerular filtration rate; LA = left atrial; LVEF = left ventricular ejection fraction; SR = sinus rhythm; VKA = vitamin K antagonist.

### RAA inflammation is associated with endothelial activation, remodeling, fibrosis, and increased SGLT2 and GLP-1R expression

**Figure 1:**
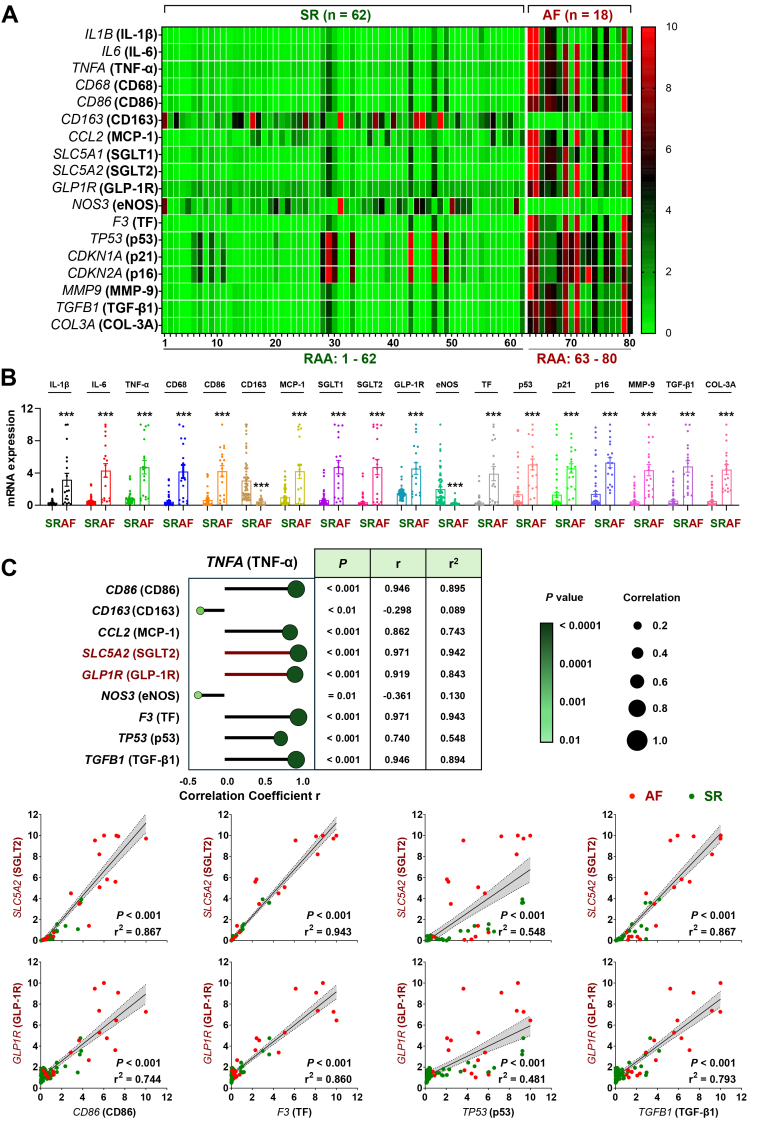
Gene Expression Analysis of Atrial Remodeling Markers, SGLT2, and GLP1R in RAAs of Patients With and Without AF (A) Heatmap of target gene expression in 80 right atrial appendages (RAAs) from 18 patients with histories of atrial fibrillation (AF) and 62 with no AF, labeled as sinus rhythm (SR). Data are shown as relative abundance represented by color (green, lower abundance; red, higher abundance) as indicated in the scale. (B) Messenger RNA (mRNA) expression levels of target genes in SR patients compared with AF patients. (C) Pearson correlations between TNF-α, sodium-glucose cotransporter 2 (SGLT2), glucagon-like peptide 1 receptor (GLP-1R), and other target gene expression levels in the 80 RAA specimens. Data are shown as mean ± SEM. *∗∗∗P* < 0.001 vs SR using multiple Student’s *t*-tests with Benjamini-Hochberg correction for false discovery rate.

RAA samples from the 80 study patients revealed marked heterogeneity in messenger RNA expression of genes associated with inflammation (*IL1B*, *IL6*, and *TNFA*), macrophages (*CD68*, *CD86*, and *CD163*), endothelial activation (*CCL2* and *NOS3*), senescence (*CDKN1A*, *CDKN2A*, and *TP53*), fibrosis (*MMP9*, *TGFB1*, and *COL3A1*), and thrombosis (*F3*), as well as SLC5A1 (*SGLT1*), SLC5A2 (*SGLT2*), and GLP1R (*GLP-1R*) (Figure 1A). In patients with histories of AF (n = 18), *NOS3* and *CD163* expression were reduced compared with patients without AF, whereas the other inflammatory, endothelial activation, fibrosis, and prothrombotic genes, together with *SLC5A1*, *SLC5A2*, and *GLP1R*, were increased (Figure 1B). Similar findings were observed in the IPTW-weighted cohort (Supplemental Table 1).

Across all RAA samples, TNFA expression positively associated with *CD86*, *CCL2*, *F3*, *TP53*, *TGFB1*, *SLC5A2*, and *GLP1R* and negatively correlated with *NOS3* and *CD163* (Figure 1C). Both SGLT2 and GLP-1R messenger RNA expression positively correlated with *CD86*, *F3*, *TP53*, and *TGFB1* expression (Figure 1C). Univariable analysis identified age ≥75 years, diabetes, AF, and chronic kidney disease (estimated glomerular filtration rate <50 mL/min/1.73 m^2^) as positive correlates of *TNFA*, *SLC5A2*, and *GLP1R* expression, whereas diabetes, AF, and hypertension were negatively associated with *NOS3* levels (Table 2). Multivariable analysis confirmed independent positive associations of age ≥75 years, diabetes, AF, and chronic kidney disease with *TNFA* and *SLC5A2* and of diabetes, AF, and chronic kidney disease with *GLP1R*, together with negative associations between diabetes and AF and *NOS3* expression (Table 3). Primer sequences for real-time quantitative PCR analysis are provided in (Supplemental Table 2).

**Table 2:** Univariable Analysis of Clinical Factors Associated With Markers

	TNF-α			SGLT2			GLP-1R			eNOS		
	*r*	*r*^2^	*P* Value	*r*	*r*^2^	*P* Value	*r*	*r*^2^	*P* Value	*r*	*r*^2^	*P* Value
Age ≥75 y	0.47	0.22	0.001	0.44	0.19	<0.001	0.39	0.15	0.001	−0.17	0.03	0.13
Male	0.06	0.004	0.60	0.08	0.006	0.51	−0.09	0.007	0.46	−0.07	0.005	0.53
Hypertension	0.19	0.04	0.10	0.20	0.04	0.08	0.16	0.03	0.16	−0.28	0.08	0.01
Dyslipidemia	0.11	0.01	0.36	0.08	0.006	0.50	0.06	0.004	0.61	−0.11	0.01	0.33
Diabetes	0.25	0.06	0.03	0.26	0.07	0.02	0.25	0.06	0.03	−0.29	0.08	0.01
Body mass index	−0.17	0.03	0.13	−0.10	0.009	0.40	−0.06	0.004	0.60	−0.22	0.05	0.05
Smoking	−0.04	0.002	0.72	−0.05	0.002	0.69	−0.11	0.01	0.34	0.12	0.02	0.29
Vascular pathology	0.09	0.008	0.44	0.08	0.006	0.52	−0.01	0.0001	0.93	−0.03	0.001	0.80
AF	0.71	0.50	0.001	0.70	0.49	0.001	0.66	0.44	0.001	−0.37	0.14	0.001
LAVI >34 mL/m^2^	0.16	0.03	0.18	0.13	0.07	0.31	−0.07	0.005	0.56	−0.15	0.02	0.23
LVEF <60%	0.004	0.000	0.97	−0.04	0.001	0.75	−0.03	0.001	0.77	0.02	0.000	0.89
eGFR <50 mL/min/1.73 m^2^	0.41	0.17	0.001	0.42	0.17	0.001	0.51	0.26	0.001	−0.02	0.000	0.86

Reverse transcriptase quantitative polymerase chain reaction measurements; 80 right atrial appendages.

eNOS = endothelial nitric oxide synthase; GLP-1R = glucagon-like peptide 1 receptor; LAVI = left atrial volume index; SGLT2 = sodium-glucose cotransporter 2; other abbreviations as in Table 1.

**Table 3:** Multivariable Analysis of Clinical Factors Associated With Markers (n = 80 Right Atrial Appendages)

	TNF-α			SGLT2			GLP-1R			eNOS		
	*r*	*r*^2^	*P* Value	*r*	*r*^2^	*P* Value	*r*	*r*^2^	*P* Value	*r*	*r*^2^	*P* Value
Age ≥ 75 y	0.19	0.04	0.03	0.20	0.04	0.02	0.14	0.02	0.10			
Hypertension										−0.19	0.04	0.07
Diabetes	0.22	0.05	0.004	0.25	0.06	0.001	0.25	0.06	0.001	−0.25	0.06	0.02
AF	0.58	0.34	0.001	0.56	0.32	0.001	0.51	0.26	0.001	−0.34	0.12	0.001
eGFR < 50 mL/min/1.73 m^2^	0.20	0.04	0.14	0.20	0.04	0.01	0.32	0.10	0.001			

Univariable parameters with *P* < 0.05 were included in the multivariable analysis. Confounding parameters were excluded (CHA_2_DS_2_-VASc score and anticoagulant agent application).

Abbreviations as in Tables 1 and 2.

For protein-level validation, samples were first stratified according to median *TNFA* messenger RNA expression into low- and high-TNF-α groups (n = 40 each). All RAA samples from patients with AF (n = 18) clustered in the high-TNF-α group, whereas samples from SR patients were distributed across both groups with 65% (40 samples) in the low-TNF-α group and 35.5% (22 samples) in the high-TNF-α group (Table 4). Within the high-TNF-α group, AF patients were older and exhibited higher LA volume index, lower left ventricular ejection fraction, and more frequent direct oral anticoagulant agent use, whereas SR patients had a higher prevalence of diabetes (Table 4). On the basis of these characteristics, 3 subgroups were defined: low TNF-α-SR, high TNF-α-SR, and high TNF-α-AF.

**Table 4:** Patient Demographic Characteristics Classified According to Median TNF-α

	Low TNF-α SR (n = 40)	High TNF-α (n = 40)		
		SR (n = 22)	AF (n = 18)	*P* Value
Age, y	64.0 (56.0-71.0)	70.0 (55.6-77.0)	76.0 (61.6-78.0)	**0.012**
Age ≥75 y	7 (17.5)	2 (9.1)	10 (55.6)	**0.001**
Male	32 (80.0)	16 (72.7)	16 (88.9)	0.20
Hypertension	18 (45.0)	14 (63.6)	13 (72.2)	0.56
Dyslipidemia	19 (47.5)	10 (45.5)	10 (55.6)	0.53
Diabetes	1 (2.5)	9 (54.6)	3 (16.7)	0.014
Smoking	14 (35.0)	6 (36.4)	2 (11.1)	0.067
Body mass index, kg/m^2^	26.4 (23.2-29.2)	27.1 (24.5-30.4)	25.0 (22.8-28.0)	0.20
History of stroke	0 (0.0)	0 (0.0)	1 (5.6)	0.27
Vascular pathology	7 (17.5)	6 (27.3)	6 (33.3)	0.68
Paroxysmal AF	0 (0.0)	0 (0.0)	14 (77.8)	**0.001**
Persistent AF	0 (0.0)	0 (0.0)	4 (22.2)	**0.02**
CHA_2_DS_2_-VASc score				
0 or 1	34 (85.0)	11 (50.0)	7 (38.9)	0.48
2 or 3	6 (15.0)	8 (36.4)	8 (44.4)	0.60
≥4	0 (0.0)	3 (13.6)	3 (16.7)	0.80
LA volume index, mL/m^2^	34.0 (25.0-43.5)	34.5 (24.7-49.8)	49.0 (33.0-61.0)	**0.025**
LA area, cm^2^	21.5 (17.0-25.5)	21.0 (18.0-26.0)	25.0 (20.0-31.0)	0.052
Aortic stenosis	8 (20.0)	8 (36.4)	5 (27.8)	0.67
LVEF, %	64.0 (60.8-67.3)	65.0 (61.5-70.0)	60.0 (58.0-63.5)	**0.041**
LVEF <60%	8 (20)	3 (13.6)	6 (33.3)	0.14
Creatinine clearance, mL/min	75.0 (68.0-89.5)	74.0 (59.8-85.0)	76.9 (66.0-96.0)	0.23
eGFR <50 mL/min/1.73 m^2^	1 (2.5)	1 (4.5)	1 (5.5)	0.86
Statins	17 (42.5)	8 (36.4)	11 (61.1)	0.12
Aldosterone antagonists	0 (0.0)	1 (4.6)	2 (11.1)	0.43
ACE inhibitors	8 (20.0)	5 (22.7)	4 (22.2)	0.97
AT1R antagonists	8 (20.0)	5 (22.7)	8 (44.4)	0.14
DOACs	2 (5.0)	2 (9.1)	10 (55.6)	**0.001**
VKAs	2 (5.0)	1 (9.1)	4 (22.2)	0.093

Values are median (Q1-Q3) or n (%). Demographic characteristics are presented for low- and high-TNF-α groups. Statistical comparisons were performed between SR and AF patients within the high-TNF-α group using the chi-square test for categorical variables and the Mann-Whitney *U* test for continuous variables. *P* values in boldface type denote statistical significance.

Abbreviations as in Table 1.

**Figure 2:**
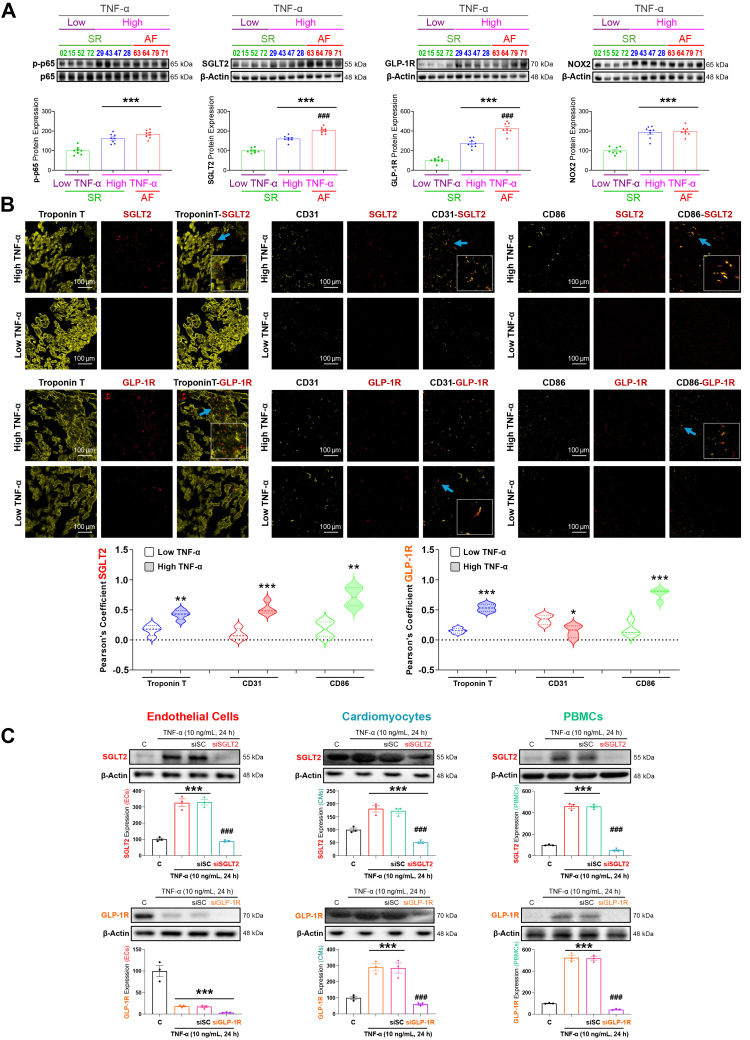
Inflammation-Associated SGLT2 and GLP-1R Expression in Human RAAs and Cardiac Cell Types RAA samples were grouped according to TNF-α expression: low TNF-α (below median); high TNF-α, including only patients in SR; and high TNF-α, including only patients with histories of AF. (A) Protein expression of p-p65, SGLT2, GLP-1R, and NOX2 shown as representative immunoblots (n = 4 per group) with corresponding quantification (n = 8 per group). (B) RAA sections with low or high TNF-α expression costained for SGLT2 or GLP-1R (red), troponin T, CD31, or CD86 (yellow). Colocalization was quantified using the Pearson correlation coefficient. (C) SGLT2 and GLP-1R expression was evaluated in human coronary artery endothelial cells (ECs), cardiomyocytes (CMs) (AC16), and peripheral blood mononuclear cells (PBMCs) after 48 hours of small interfering RNA (siRNA)–mediated knockdown (vs scrambled siRNA control) followed by TNF-α stimulation (10 ng/mL, 24 hours) and western blot analysis. Data are presented as mean ± SEM. Statistical significance was determined using 1-way analysis of variance followed by Tukey’s post hoc test in (A) (*∗P* < 0.05 vs low TNF-α; ^#^*P* < 0.05 vs high TNF-α-SR) and (C) (*∗P* < 0.05 vs control; ^#^*P* < 0.05 vs TNF-α-stimulated cells). For B, comparisons between low- and high-TNF-α groups were performed using multiple Student’s *t*-tests with Benjamini-Hochberg correction for false discovery rate (*∗P* < 0.05). *∗P* < 0.05, *∗∗P* < 0.01, *∗∗∗P* < 0.001, and ^###^*P* < 0.001. Abbreviations as in Figure 1.

Overall, RAA samples from the high-TNF-α group exhibited lower endothelial nitric oxide synthase expression and higher p-p65, SGLT2, GLP-1R, NOX2, VCAM-1, tissue factor, p53, p21, MMP-9, TGF-β1, and collagen-3a compared with those from the low-TNF-α group (Figure 2A, Supplemental Figure 1). Within the high-TNF-α group, SGLT2, GLP-1R, tissue factor, MMP-9, TGF-β1, and collagen-3a were further elevated in RAA samples from AF patients compared with SR patients. Additionally, Sirius red staining confirmed increased collagen deposition in the high-TNF-α group, with the highest fibrosis levels observed in patients with AF (Supplemental Figure 1).

Collectively, these data suggest an atrial inflammatory phenotype associated with AF, characterized by endothelial activation, senescence, increased tissue factor expression, structural remodeling and fibrosis, and increased SGLT2 and GLP-1R expression.

### Inflammation-related cellular expression of SGLT2 and GLP-1R in human RAA tissue and cardiac cells

Next, to delineate the cell-specific origins of the increased SGLT2 and GLP-1R expression in inflamed RAA tissue, we performed comprehensive immunofluorescence analysis. RAA samples from the high-TNF-α group showed strong colocalization of SGLT2 with CD31 (an endothelial marker) and CD86 (a proinflammatory macrophage marker). CD86 also colocalized with CCR2, suggesting that these cells most likely represent proinflammatory recruited macrophages in the inflamed RAA (Supplemental Figure 2). SGLT2 exhibited weaker colocalization with troponin T (a cardiomyocyte marker). Additionally, immunofluorescence stains of RAA samples from the low-TNF-α group qualitatively showed less SGLT2 and CD86 expression compared with the high-TNF-α group (Figure 2B).

In contrast to SGLT2 expression, GLP-1R expression predominantly colocalized with troponin T and CD86 in the RAA samples from the high-TNF-α group, whereas colocalization with CD31 was minimal. GLP-1R expression was qualitatively lower in the low-TNF-α group, in which it predominantly colocalized with CD31 (Figure 2B).

To further support our findings, we reanalyzed a publicly available human atrial single-nucleus RNA sequencing data set (SCP2489). This confirmed cell type–specific expression of SLC5A2 (SGLT2) and GLP1R in the atrium, including endothelial cells, immune cells, and cardiomyocytes (Supplemental Figure 3). Notably, in the original study, *SLC5A2* or *GLP1R* were not differentially expressed between patients with AF vs control subjects, likely because of low abundance transcript levels and potential effects from collecting samples from postmortem donors.^13^ In contrast, our RAA samples were collected from patients at the time of cardiac surgery. Additionally, we primarily used protein, as opposed to messenger RNA levels, to assess atrial SGLT2 and GLP-1R expression levels in relation to inflammation, disease, and cell type.

To further validate the cell-specific patterns of SGLT2 and GLP-1R expression observed in our inflamed RAA samples, we assessed their expression in human coronary artery endothelial cells, cardiomyocytes (AC16 cells), and peripheral blood mononuclear cells (PBMCs) (from healthy donors), following TNF-α stimulation. In human coronary artery endothelial cells, TNF-α markedly up-regulated SGLT2 expression, an effect abolished by SGLT2 knockdown. Conversely, GLP-1R expression was reduced after TNF-α stimulation and by GLP-1R knockdown (Figure 2C). In human cardiomyocytes and PBMCs, TNF-α increased both SGLT2 and GLP-1R expression, with responses attenuated by respective knockdown (Figure 2C). Efficient small interfering RNA–mediated silencing of SGLT2 and GLP-1R was confirmed at the messenger RNA level (Supplemental Figure 4).

### ROS formation in inflamed RAA is attenuated by SGLT2 inhibition

**Figure 3:**
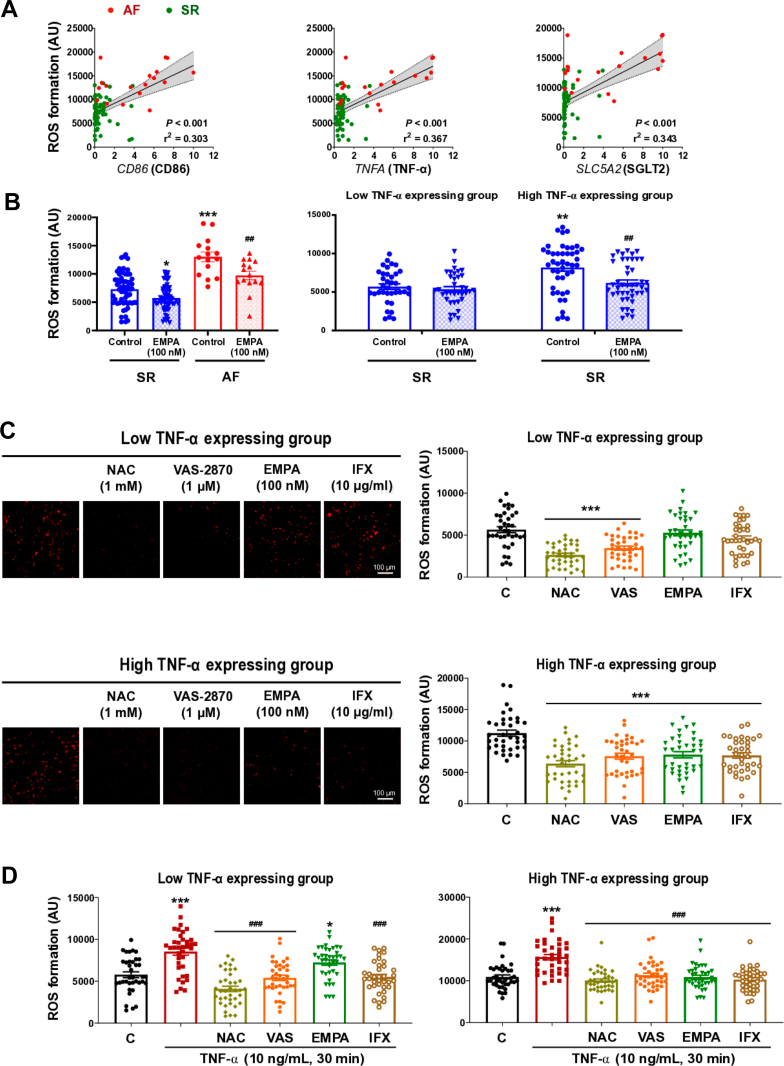
Antioxidant Effects of SGLT2 Inhibition in RAA Tissue From Patients With or Without AF (A) Pearson correlations between ROS and CD86, TNF-α, and SGLT2 gene expression in 80 RAA samples (*r*^2^ is the coefficient of determination). (B) Effect of empagliflozin on reactive oxygen species (ROS) formation in RAAs from AF vs SR patients, with the latter group also subcategorized into low- vs high-TNF-α-expressing groups. (C) ROS formation measured in low- vs high-TNF-α-expressing RAAs under basal conditions and after treatment with *N*-acetylcysteine (NAC; 1 mM, 2 hours), VAS-2870 (VAS; a nicotinamide adenine dinucleotide phosphate oxidase inhibitor, 1 μM), empagliflozin (EMPA; 100 nM), or infliximab (IFX; a TNF-α-neutralizing antibody, 30 ng/mL) for 30 minutes prior to dihydroethidium staining. (D) RAA sections exposed to TNF-α with or without inhibitors. Data are shown in arbitrary units (AU) as mean ± SEM. ∗*P* < 0.05 vs control (C) and SR control (A) and ^#^*P* < 0.05 vs AF control B and TNF-α-stimulated sections (D), analyzed using 1-way or 2-way analysis of variance (B) followed by Tukey’s test. *∗P* < 0.05, *∗∗P* < 0.01, *∗∗∗P* < 0.001, ^##^*P* < 0.01, and ^###^*P* < 0.001. Abbreviations as in Figure 1.

As inflammation is a key driver of oxidative stress in the heart and vasculature,^14^ we examined ROS levels in the RAA specimens. Across all samples, ROS levels were positively correlated with CD86, TNF-α, and SGLT2 expression (Figure 3A). RAA samples from AF patients, which all clustered in the high-TNF-α group, exhibited higher ROS levels compared with SR patients. Importantly, treatment with empagliflozin reduced the elevated ROS levels in RAA samples from AF patients (Figure 3B). In RAA samples from SR patients, empagliflozin had no effect on ROS formation in those in the low-TNF-α subgroup but reduced ROS levels in those that clustered in the high-TNF-α subgroup (Figure 3B).

These findings suggest that elevated ROS formation in the atria is related to heightened inflammation and SGLT2 activity. Indeed, in the low-TNF-α group, basal ROS formation (5,654 arbitrary units) was reduced by 53% by *N*-acetylcysteine and 39% by the nicotinamide adenine dinucleotide phosphate oxidase inhibitor VAS-2870 but was unaffected by empagliflozin or the TNF-α-neutralizing antibody infliximab (Figure 3C). In contrast, in the high-TNF-α group, in which basal ROS levels were higher at 11,239 arbitrary units, these were reduced not only by *N*-acetylcysteine (43%) and VAS-2870 (33%) but also by empagliflozin (31%) and infliximab (26%) (Figure 3C). Univariable analysis also showed that age ≥75 years, hypertension, diabetes, AF, and LA volume index (LA volume index >34 mL/m^2^) were positively associated with basal ROS, and multivariable analysis identified diabetes, AF, and LA volume index as independent correlates (Table 5).

**Table 5:** Univariable and Multivariable Analysis of Clinical Factors Associated With Markers

	Univariate Analysis ROS Levels			Multivariate Analysis ROS Levels		
	*r*	*r*^2^	*P* Value	*r*	*r*^2^	*P* Value
Age ≥75 y	0.30	0.09	0.012	0.10	0.009	0.29
Male	−0.16	0.03	0.17			
Hypertension	0.26	0.07	0.03	0.14	0.02	0.13
Dyslipidemia	0.10	0.01	0.41			
Diabetes	0.41	0.17	0.001	0.34	0.11	0.001
Body mass index	−0.01	0.000	0.93			
Smoking	−0.22	0.05	0.062			
Vascular pathology	0.11	0.012	0.36			
AF	0.61	0.37	0.001	0.51	0.26	0.001
LAVI >34 mL/m^2^	0.37	0.14	0.003	0.24	0.06	0.008
LVEF <60%	0.02	0.000	0.90			
eGFR <50 mL/min/1.73 m^2^	0.23	0.05	0.056			

ROS measurements; 80 right atrial appendages. Univariable parameters with *P* < 0.05 were included in the multivariable analysis. Confounding parameters were excluded (CHA_2_DS_2_-VASc score and anticoagulant agent application).

ROS = reactive oxygen species; other abbreviations as in Tables 1 and 2.

To further assess the inflammatory contribution to ROS formation in the RAA, cryosections from both groups were exposed to TNF-α prior to ROS measurement. In the low-TNF-α group, recombinant TNF-α increased ROS to 8,532 arbitrary units, which could be prevented by *N*-acetylcysteine, VAS-2870, and infliximab but not empagliflozin (Figure 3D). In the high-TNF-α group, recombinant TNF-α exposure further increased ROS by about 1.5-fold to 15,655 arbitrary units, which was effectively reduced by *N*-acetylcysteine (36%), VAS-2870 (27%), empagliflozin (31%), and infliximab (26%) (Figure 3D).

### GLP-1Ra attenuate inflammation-related atrial ROS formation via cell type-specific effects

**Figure 4:**
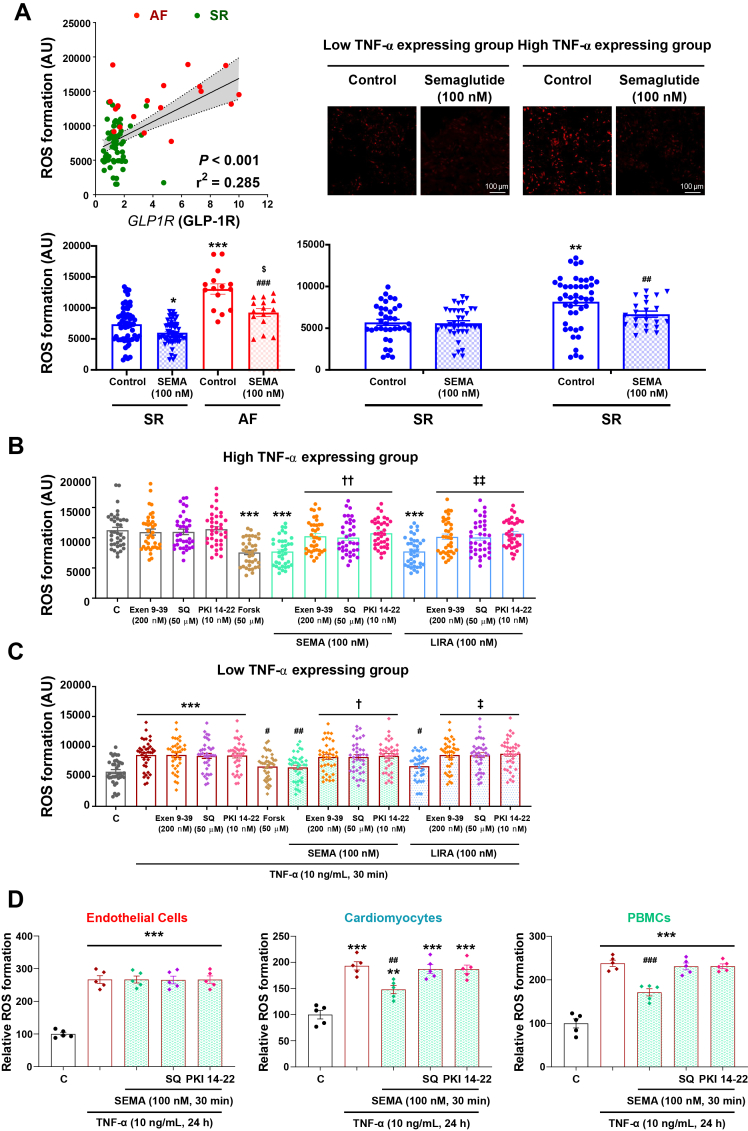
Antioxidant Effects of GLP-1R Agonists in RAA Tissue From Patients With or Without AF (A) Pearson correlation between ROS and GLP-1R gene expression in 80 RAA samples (*r*^2^ is the coefficient of determination). Effect of semaglutide (SEMA) on ROS formation in RAAs from AF vs SR patients, with the group also subcategorized into low- vs high-TNF-α-expressing groups. Dihydroethidium (DHE) staining was done 30 minutes after treatment. (B) High-TNF-α-expressing RAAs treated with exendin 9-39 (Exen 9-39; a GLP-1R antagonist), SQ22536 (SQ; an adenylyl cyclase inhibitor), PKI (14-22) (a protein kinase A inhibitor), forskolin (Forsk; an adenylyl cyclase activator), and SEMA or liraglutide (LIRA; 100 nM), with or without inhibitors, for 30 minutes before DHE staining. (C) Low TNF-α-expressing RAAs exposed to TNF-α in the absence or presence of inhibitors. ROS levels measured ECs, AC16 CMs, and PBMCs pretreated with SEMA (100 nM) with or without SQ22536 or PKI for 30 minutes prior to TNF-α stimulation (10 ng/mL, 24 hours). Data are presented as mean ± SEM (AU). ∗*P* < 0.05 vs respective control, #*P* < 0.05 vs AF control A or TNF-α-stimulated RAAs C or cells (D), $*P* < 0.05 vs SEMA SR A, †*P* < 0.05 vs SEMA (B and C), and ‡*P* vs LIRA (B and C) analyzed using 1-way or 2-way analysis of variance A with Tukey’s test. ∗∗*P* < 0.01, ∗∗∗*P* < 0.001, #*P* < 0.05, ##*P* < 0.01, ###*P* < 0.001, †*P* < 0.05, ††*P* < 0.01, ‡*P* < 0.05, and ‡‡P < 0.01. Abbreviations as in Figure 1, Figure 2, Figure 3.

Although RAA inflammation positively associated with GLP-1R expression (Figures 1 and 2), prior studies have reported antioxidant effects of GLP-1Ra in cardiovascular cells.^15^ To further examine this, we assessed the effects of GLP-1R activation on RAA ROS formation. GLP-1R expression positively correlated with ROS levels in RAA samples (*r*^2^ = 0.285) (Figure 4A). Semaglutide reduced ROS levels in RAA samples from both AF and SR patients (Figure 4A). However, within the SR patient group, semaglutide had no effect on lowering ROS formation in the low-TNF-α subgroup, but effectively reduced ROS in the high-TNF-α subgroup (Figure 4A).

Given the known effects of GLP-1Ra on cyclic adenosine monophosphate (cAMP)–dependent protein kinase A (PKA) signaling, we tested whether this downstream signaling mechanism may be involved in their antioxidant effects in inflamed RAA tissue. The inhibitory effects of semaglutide and liraglutide on ROS formation were abolished by exendin 9-39 (a GLP-1R antagonist), SQ22536 (adenylyl cyclase inhibitor), and PKI (14-22) (a PKA inhibitor) (Figure 4B). Conversely, forskolin (an adenylyl cyclase activator) reduced ROS by 32%, comparable with semaglutide and liraglutide (Figure 4B).

Moreover, in the low-TNF-α group, ROS formation induced by additional recombinant TNF-α exposure was decreased by about 24% by both semaglutide and liraglutide. This effect was prevented by exendin 9-39, SQ22536, and PKI (14-22) but mimicked by forskolin (Figure 4C). Exendin 9-39, SQ22536, and PKI (14-22) alone did not affect basal ROS formation in the high-TNF-α group or the low-TNF-α group exposed to additional recombinant TNF-α (Figures 4B and 4C).

To validate these findings in relevant human cardiac cell types, ROS quantification was performed in TNF-α-stimulated human coronary artery endothelial cells, PBMCs, and AC16 cells. TNF-α increased ROS in all 3 cell types. In cardiomyocytes and PBMCs, TNF-α-induced ROS formation was similarly reduced by semaglutide through a cAMP/PKA-dependent pathway. However, in endothelial cells, TNF-α-induced ROS was unaffected by semaglutide (Figure 4D). Taken together, these findings suggest that GLP-1Ra can reduce inflammation-related ROS formation in atrial tissue through a cAMP/PKA-dependent pathway but with variable effects in different cell types.

### Combination of SGLT2i plus GLP-1Ra has synergistic antioxidant effects in inflamed human RAA tissue and cardiac cells

**Figure 5:**
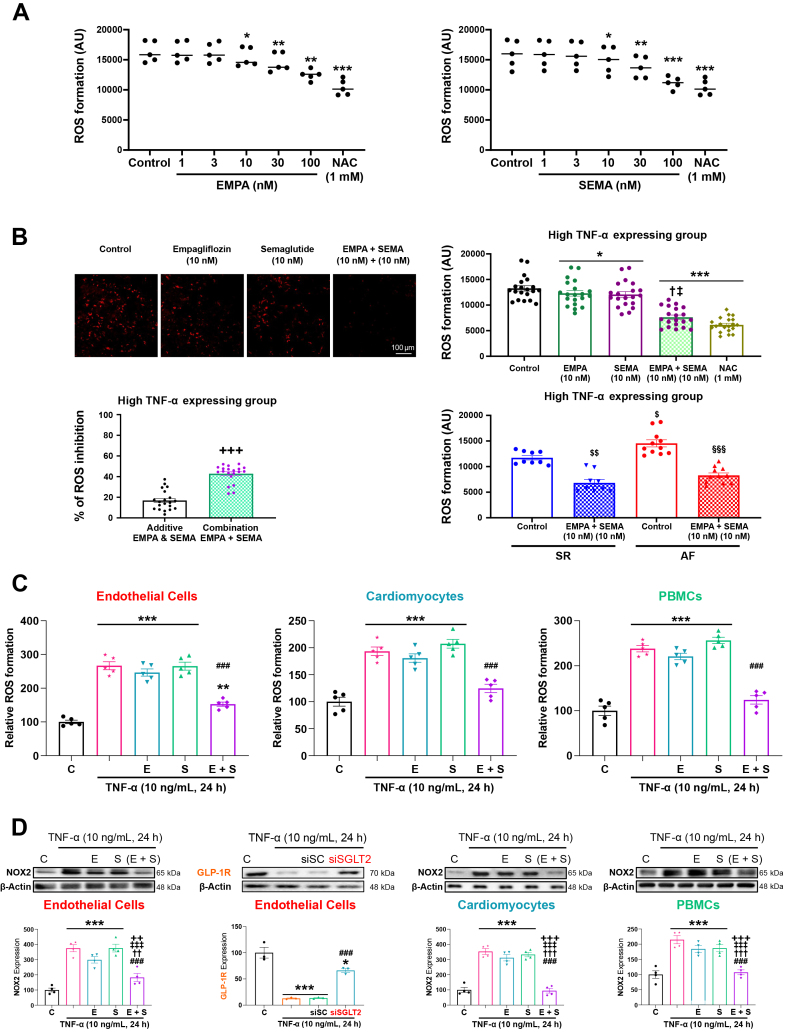
Synergistic Antioxidant and Anti-Inflammatory Effects of SGLT2 Inhibitors and GLP-1R Agonists in Human RAAs and Cardiac Cells (A) ROS formation assessed by DHE staining in high-TNF-α-expressing RAAs under basal conditions and after treatment with EMPA or SEMA (30 minutes) or NAC (2 hours). (B) In the upper quartile TNF-α-expressing RAAs, ROS levels measured after treatment with EMPA, SEMA, their combination, or NAC. Combined effect compared with the expected additive effect, and responses analyzed in SR vs AF patients. (C and D) ECs, CMs (AC16), and PBMCs pretreated with EMPA (10 nM), SEMA (10 nM), or their combination for 30 minutes or following 48 hours of gene knockdown using SGLT2 siRNAs. Scrambled siRNA (Scrbl) served as the negative control. Cells were subsequently stimulated with TNF-α (10 ng/mL) for 24 hours. ROS formation was measured by DHE staining C and target protein expression by western blot D Data are presented as mean ± SEM (AU). ∗*P* < 0.05 vs control, $*P* < 0.05 vs SR control, §*P* < 0.05 vs AF control, #*P* < 0.05 vs TNF-α-stimulated cells, †*P* < 0.05 vs EMPA, ‡*P* < 0.05 vs SEMA, and +*P* < 0.05 vs expected additive effect of EMPA plus SEMA, analyzed using 1-way analysis of variance, 2-way analysis of variance B with Tukey’s multiple-comparison test, or unpaired Student’s *t*-test (B). *∗P* < 0.05, *∗∗P* < 0.01, *∗∗∗P* < 0.001, #*P* < 0.05, ###*P* < 0.001, $*P* < 0.05, $$*P* < 0.01, §§§*P* < 0.001, †*P* < 0.05, ††*P* < 0.01, †††*P* < 0.001, ‡*P* < 0.05, ‡‡*P* < 0.01, ‡‡‡*P* < 0.001, and +++*P* < 0.001. Abbreviations as in Figure 1, Figure 2, Figure 3, Figure 4.

Because SGLT2 inhibition and GLP-1R activation both reduce inflammation-related atrial ROS formation, but exhibit different cell expression patterns and mechanisms of action, we tested whether low-dose combination therapy would enhance their effects. Dose-response curves showed that both empagliflozin and semaglutide significantly reduced ROS in the high-TNF-α group at concentrations ≥10 nM (Figure 5A). At 10 nM, empagliflozin and semaglutide reduced ROS by 6% and 6.3%, respectively, and by 24% and 30% at 100 nM, whereas *N*-acetylcysteine reduced ROS by 36% (Figure 5A).

In RAA from the upper quartile TNF-α group, empagliflozin and semaglutide at 10 nM reduced ROS by 7.2% and 9.1%, respectively. However, the combination of these 2 agents produced a markedly greater reduction of 42.4%, exceeding either agent alone and comparable with *N*-acetylcysteine, consistent with a synergistic antioxidant effect (Figure 5B). This effect was observed in both SR and AF patients within the upper quartile TNF-α group (Figure 5B).

Additionally, in our cell culture models in which recombinant TNF-α induces ROS formation, the combination of 10 nM empagliflozin plus 10 nM semaglutide reduced ROS in all 3 cell types, an effect not observed with either agent alone when dosed at 10 nM (Figure 5C). Recombinant TNF-α up-regulated NOX2, MMP-9, and TGF-β1 expression in cardiomyocytes and NOX2, CD86, and CCR2 expression in PBMCs, which were attenuated by the low-dose combination (Figure 5D, Supplemental Figure 5). In endothelial cells, TNF-α increased NOX2, VCAM-1, and p53 expression and reduced VASP phosphorylation at Ser157, effects that were attenuated by combined empagliflozin and semaglutide at 10 nM (Figure 5D, Supplemental Figure 5). Notably, SGLT2 silencing prevented TNF-α-induced GLP-1R down-regulation in endothelial cells, suggesting potential regulatory crosstalk between these proteins (Figure 5D).

## Discussion

The present findings demonstrate that patients with histories of AF exhibit a marked inflammatory phenotype in the RAA, characterized by increased proinflammatory macrophages, oxidative stress, endothelial activation, and activation of remodeling, fibrotic, and thrombotic pathways. This pathologic RAA phenotype is less pronounced in patients without AF but varies according to the level of atrial inflammation. Importantly, in this study, we identify a strong link between ROS formation in the RAA with increased SGLT2 expression, predominantly in proinflammatory macrophages and endothelial cells, and GLP-1R expression, mainly in macrophages and cardiomyocytes, that correlates with the degree of atrial inflammation. Both SGLT2i and GLP-1Ra are established first-line HF therapies with different mechanisms of action that can counteract cardiac inflammation and oxidative stress. To the best of our knowledge, our findings are the first to show that combined SGLT2i and GLP-1Ra treatment can synergistically reduce inflammation-driven oxidative stress in atrial tissue associated with atrial cardiomyopathy and AF.

### Increased SGLT2 expression in inflammation-related ROS formation in atrial cardiomyopathy

A key finding from our work is the identification of increased SGLT2 expression in the RAA of patients with AF or high levels of atrial inflammation. Immunofluorescence analysis showed that SGLT2 colocalizes predominantly with proinflammatory macrophages and endothelial cells in highly inflamed RAA tissue, which was supported by TNF-α up-regulation of SGLT2 and NOX2 in cultured human endothelial cells, cardiomyocytes, and PBMCs. Although basal SGLT2 expression is generally low in healthy cardiac cells, other have similarly shown that its expression is up-regulated by inflammatory stimuli including TNF-α, angiotensin II, thrombin, and factor Xa, via redox-sensitive signaling pathways.^16^, ^17^, ^18^, ^19^ Importantly, our data are the first to show that SGLT2i, such as empagliflozin, can directly reduce ROS formation in highly inflamed RAA tissue from AF patients.

Although further investigation into the specific mechanisms by which SGLT2i attenuate inflammation-related ROS formation in the atria is warranted, we propose that increased SGLT2 expression may sustain glucose-driven pro-oxidant signaling that promotes endothelial dysfunction and remodeling.^19^ In line with this concept, SGLT2 inhibition has been shown to attenuate sustained (but not initial) angiotensin II–induced oxidative stress in endothelial cells in parallel with SGLT2 protein expression.^18^^,^^19^ Studies in human myocardial slices from patients with HF have also shown that SGLT2 inhibition down-regulates extracellular matrix–related genes in fibroblasts and improves oxidative stress and inflammatory responses in cardiomyocytes.^20^

### Antioxidant effect of GLP-1Ra in atrial inflammation

Similar to SGLT2, we found that GLP-1R messenger RNA and protein expression in human RAA increase with inflammation severity. However, a different pattern of cell type expression was found. In highly inflamed RAA tissue, the increased GLP-1R expression predominantly colocalized with cardiomyocytes and macrophages but decreased in endothelial cells. Comparable patterns were observed in cultured endothelial cells, cardiomyocytes, and PBMCs following TNF-α stimulation.

Interestingly, similar patterns of cell type expression have been observed in apolipoprotein E–deficient mice, in which GLP-1R expression localizes to endothelial cells at early stages but shifts toward vascular smooth muscle cells and infiltrating macrophages with disease progression.^21^

Why myocytes and macrophages up-regulate GLP-1R expression in the context of increased inflammation is unclear but may represent more of a compensatory process. In our functional experiments, semaglutide and liraglutide reduced pro-oxidant signaling in highly inflamed RAAs from AF and SR patients, without affecting the basal oxidative stress in RAA tissue from SR patients with low-grade inflammation. These antioxidant effects were mediated via canonical GLP-1R cAMP-dependent PKA signaling, as shown by blockade with exendin 9-39 or adenylyl cyclase and PKA inhibitors and mimicking by forskolin. The mechanisms by which GLP-1R activation modulates ROS formation in the atria need to be defined, but could parallel similar effects in the ventricle and other organ systems in which these agents modulate redox-sensitive transcriptional programs by suppressing proinflammatory NF-κB signaling, enhancing anti-inflammatory Nrf2 activation, and reducing profibrotic remodeling.^22^, ^23^, ^24^, ^25^, ^26^, ^27^

### Synergistic effects of combined empagliflozin plus semaglutide treatment on inflammation-related oxidative stress in RAA

Although both SGLT2i and GLP-1Ra independently reduced ROS formation in highly inflamed RAA tissue, the most striking finding was the synergistic effects these agents had when used in combination. Low-dose empagliflozin combined with semaglutide synergistically reduced pro oxidant signaling in RAA and attenuated inflammatory oxidative responses in human cardiac cells. In TNF-α-stimulated cardiomyocytes, endothelial cells, and PBMCs, the combined treatment effectively restored ROS formation back to normal baseline levels. The reasons for this synergistic effect are likely multifactorial, with distinct cellular distribution of SGLT2 and GLP-1R in highly inflamed RAA tissue, together with their different mechanisms of action.

Interestingly, our in vitro work in human cardiac cells identified a potential regulatory crosstalk mechanism between SGLT2 and GLP-1R expression, which could also contribute to the synergistic effects observed with combining these agents. Specifically, we found that SGLT2 knockdown prevented TNF-α-induced down-regulation of GLP-1R in endothelial cells. This could potentially preserve GLP-1R availability under inflammatory conditions and promote nitric oxide signaling via AMPK signaling.^28^ Combined empagliflozin and semaglutide consistently prevented TNF-α-induced reduction of VASP phosphorylation at the cAMP site, an effect not observed with either agent alone. Moreover, TNF-α-induced oxidative stress in endothelial cells was not reduced by semaglutide alone. However, even at lower concentrations, cotreatment with empagliflozin prevented TNF-α-induced ROS formation, supporting the concept that SGLT2 down-regulation may enhance GLP-1R functional responsiveness and enable agonist-mediated protection in the endothelium. The precise mechanisms underlying SGLT2-GLP-1R interplay require further investigation.

Collectively, these data support potential crosstalk between SGLT2- and GLP-1R-related pathways that could potentially reduce inflammation-related atrial oxidative stress associated with AF. In line with this concept, emerging clinical data suggest that combined SGLT2i and GLP-1Ra therapy reduces the risk for major adverse cardiovascular events in patients with type 2 diabetes by about 30% compared with either class alone.^29^^,^^30^ Further work is needed to determine if this combined strategy may have similar benefits in AF.

### Study limitations

This study was conducted in a relatively small cohort of cardiac surgery patients (n = 80). IPTW was applied to adjust for differences in baseline characteristics between groups. However, the use of a relatively lenient threshold (standardized mean difference <0.25) to assess covariate balance after weighting represents a limitation, as residual confounding cannot be completely excluded. All patients were in SR at the time of surgery, which may underestimate the impact of persistent AF, and silent or asymptomatic AF cannot be ruled out.

Although analyses focused on the RAA, AF predominantly involves the LA and LA appendage. Nevertheless, remodeling and fibrosis may affect both atria. The present work primarily addressed structural remodeling and did not evaluate electrophysiological alterations or the effects of combined SGLT2i and GLP-1Ra therapy on electrical remodeling.

Local TNF-α concentrations within atrial tissue were not quantified. The concentrations of empagliflozin and semaglutide used experimentally were lower than typical clinical plasma levels, and systemic concentrations may not reflect atrial tissue exposure. The combined low-dose treatment (10 nM each) was selected on the basis of concentration-response curves for ROS inhibition in inflamed RAA and may not directly correspond to clinically achieved levels.

Although cell-specific colocalization of SGLT2 and GLP-1R was demonstrated in atrial tissue, further immunophenotyping and in vitro studies under conditions better reflecting chronic low-grade inflammation are warranted. Furthermore, detailed mechanistic investigation of GLP-1R activation under conditions of SGLT2 down-regulation will require dedicated future studies.

## Conclusions

The present findings demonstrate that AF is strongly linked to increased inflammation and SGLT2 and GLP-1R expression in atrial tissue. Increased levels of SGLT2 and GLP-1R in inflamed RAA are associated with markers of adverse remodeling and oxidative stress. The synergistic antioxidant effect of the combination of an SGLT2i plus a GLP-1Ra may represent a novel therapeutic strategy to reduce inflammation-related atrial remodeling and oxidative stress in AF.

Perspectives **COMPETENCY IN MEDICAL KNOWLEDGE:** AF is associated with an inflammation-related atrial phenotype, characterized by increased endothelial dysfunction, proinflammatory macrophages, oxidative stress, and fibrotic remodeling. This study identifies up-regulation of SGLT2 and the GLP-1R in the atrial myocardium and demonstrates that targeting these pathways attenuates inflammatory and oxidative responses. These findings support the concept of AF as an atrial cardiomyopathy and suggest that cardiometabolic therapies could modulate the structural substrate underlying AF beyond rhythm control alone.

**TRANSLATIONAL OUTLOOK:** This study identifies inflammation-associated increases in SGLT2 and GLP-1R signaling as key contributors to oxidative stress, endothelial dysfunction, and atrial remodeling in AF. The observed synergistic effects of combined SGLT2 inhibition and GLP-1R activation on atrial oxidative stress and inflammation provide a rationale for future translational investigation. Prospective preclinical and clinical studies are warranted to determine whether dual therapy with an SGLT2i and a GLP-1Ra can prevent or slow AF-associated atrial cardiomyopathy and improve rhythm control and cardiovascular outcomes.

### Data availability

All data supporting the findings of this study are incorporated within the paper and its supplementary materials. Additional raw data are available from the corresponding author upon reasonable request, subject to ethical and privacy considerations.

## Funding Support and Author Disclosures

This project was supported by Groupe pour l’Enseignement, la Prévention et la Recherche Cardiovasculaire en Alsace and by Boehringer Ingelheim Pharma. Dr Kikuchi has received a grant from the Japan Heart Foundation/Bayer Yakuhin Research Grant Abroad. Dr Gong was supported by the Basic Science Research Program through the National Research Foundation of Korea, funded by the Ministry of Education (IRIS RS-2025-25436621). Dr Morel has received grants from AstraZeneca, Medtronic, and Boehringer Ingelheim. Dr Schini-Kerth has received grants from Boehringer Ingelheim, Schwabe, and Servier. Dr Pieper is an employee of Boehringer Ingelheim. All other authors have reported that they have no relationships relevant to the contents of this paper to disclose.
