# Supplementary material for: Synergistic Effects of SGLT2 Inhibitors and GLP-1R Agonists on Inflammation-Associated Oxidative Stress in Atrial Fibrillation
Source: JACC Basic Transl Sci. 2026 Jun 6;11(7):101587. doi: 10.1016/j.jacbts.2026.101587 (PMC13266112; doi:10.1016/j.jacbts.2026.101587)
Supplement: Supplemental Material 1 [file mmc1.docx]

**Supplemental Appendix**

**Supplemental Methods:** Page 2

**Supplemental Tables:**

Supplemental Table 1: page 8

Supplemental Table 2: page 10

**Supplemental Figures:**

Supplemtanl Figure 1: page 12

Supplemtanl Figure 2: page 14

Supplemtanl Figure 3: page 15

Supplemtanl Figure 4: page 17

Supplemtanl Figure 5: page 18

**References:** Page 19

**Supplemental Methods:**

Unless indicated, all chemicals and solvents were from Sigma-Aldrich (St Quentin Fallavier, France). TNF-α was purchased from PeproTech (Neuilly-Sur-Seine, France). Empagliflozin was provided by Boehringer Ingelheim Pharma GmbH & Co KG (Biberach an der Riss, Germany) and Semaglutide (NNC 0113-02) and Liraglutide (NNC 0090-1170) by Novo Nordisk (Copenhagen, Denmark). Infliximab was from Thermofisher (Illkirch, France). Mouse monoclonal anti-eNOS (1:1000; ab76193), anti-ICAM-1 (1:1000; ab171123), anti-TGF-β (1:1000; ab27969), anti-MMP-2 (1:1000; ab86607), anti-NOX2 (1/4500; ab129068**)** and anti-GAPDH (1:10000; ab8245) and rabbit monoclonal anti-p53 (1:1000; ab61241) and anti-Troponin T (1:250; ab209813) were purchased from Abcam (Cambridge, UK). Mouse monoclonal anti-TF (1:100 TF; 4509) from Sekisui Diagnostic (Darmstadt, Germany), anti-CCR2 (1/500; MABF-299) from EMD Millipore Sigma, anti-p21 (1:100; SC-397) and anti-CD31 (1:100; 550300) from BD Bioscience Pharmingen (East Rutherford, New Jersey, USA), anti-p16 (1:200; sc-390485), anti-GLP-1R (1:1000; sc-390774 and anti-SGLT2 Alexa Fluor 647 (1:250 ; sc-393350) from Santa Cruz Biotechnology (Dallas, Texas, USA). anti-phospho NF-kB (p-p65, 1:1000; 3033S) and anti-CD86 (1:250; 918825) from Cell Signaling (Saint-Cyr, France), and rabbit polyclonal anti-SGLT1 (1:1000; agt-031) and anti-SGLT2 (1:1000; agt-032) from Alomone lab (Jerusalem, Israel). Goat anti-mouse immunoglobulin G coupled to CF 633 (1:250, Alexa Fluor 633 conjugate), goat anti-mouse immunoglobulin G coupled to CF 488 (1:250, Alexa Fluor 488 conjugate) and goat anti-rabbit immunoglobulin G coupled to CF 633 (1:250, Alexa Fluor 633 conjugate) were purchased from Invitrogen (Strasbourg, France).

**Public single-nucleus RNA-sequencing analysis**
Processed human atrial single-nucleus RNA-sequencing data were downloaded together with metadata and cell-type annotations^1^. All available samples, including atrial fibrillation and control individuals, were included. UMAP embeddings and dot plots for SLC5A2 (SGLT2) and GLP1R (GLP-1R) were generated directly using the Broad Single Cell Portal interface and are shown in Supplementary Figure 3. As reported in the original publication (Supplementary Data 4; Hill MC et al., Nat Commun. 2024;15:10002) cell-type–specific differential expression analysis did not identify SLC5A2 or GLP1R as significantly differentially expressed genes.

**In vitro experiments**

**Human cardiomyocyte culture**

AC16 human cardiomyocyte cells (obtained from PluriCell East platform at the Institute of Genetics and Molecular and Cellular Biology, IGBMC, Strasbourg, France) were cultured in DMEM/F12 medium (Invitrogen) supplemented with 10% fetal bovine serum (FBS; EMD Millipore, Cat. No. ES-009-B), penicillin (100 IU/mL), streptomycin (100 IU/mL), fungizone (2.5 μg/mL), and L-glutamine (2 mM; all from Lonza, St. Quentin en Yvelines, France). Cells were maintained in a humidified incubator at 37 °C with 5 % CO₂ to promote optimal adhesion and growth.

**Human coronary artery endothelial cell culture**

Human coronary artery endothelial cells (hCAECs, #CC-2858, Lonza) were cultured in EBM®-2 Basal Medium (CC-3156, Lonza) supplemented with the EGM®-2 MV BulletKit (CC-4147, Lonza), containing 5 % FBS, 0.2 µg/mL hydrocortisone, 4 ng/mL hEGF-B, 2 ng/mL VEGF, 5 ng/mL R3-IGF-1, 75 µg/mL ascorbic acid, 10 ng/mL hEGF, 30 µg/mL gentamicin, and 15 ng/mL amphotericin B. Cells were maintained in a humidified incubator at 37 °C with 5 % CO₂.

**Human peripheral blood mononuclear cell isolation and culture**

Blood samples were collected from healthy donors aged 20–30 years with no history of cardiovascular disease in EDTA tubes at the University Hospital of Strasbourg, France. Whole blood was gently layered over Ficoll (CMSMSL01-01, Eurobio Scientific) at a 1:1 ratio and centrifuged at 1000 × g for 30 min at room temperature. The peripheral blood mononuclear cell (PBMC) layer was collected, resuspended in VersaLyse lysing solution (A09777, Eurobio Scientific; 250 µL per 10 mL of blood), and incubated for 15 min in the dark. Cells were then centrifuged at 500 × g for 15 min at room temperature. The resulting PBMC pellet was quantified and cultured in RPMI-1640 medium (Invitrogen) supplemented with 10% FBS, penicillin (100 IU/mL), streptomycin (100 IU/mL), fungizone (2.5 μg/mL), and L-glutamine (2 mM) in a humidified incubator at 37 °C with 5 % CO₂.

**Cell stimulation**

For all experiments, cells were incubated in serum-free culture medium for 2 h, followed by treatment with a pharmacological modulator for 30 min. Subsequently, cells were stimulated with TNF-α (10 ng/mL) for 24 h while untreated cells served as controls.

**SGLT2 and GLP-1R siRNA transfection**

Cells were transfected with human siRNA (50 nM) targeting SGLT2 (pre-designed siRNA, SI00725137, Qiagen) or GLP-1R (custom-designed siRNA; sense: GGACGCACCUCAUUCUACA; antisense: CCUGCGUGGAGUAAGAUGU; Sigma-Aldrich), along with a negative control (scrambled siRNA, SI03650318, Qiagen).Transfections were performed in OPTI-MEM™ medium (#31985070, Gibco) using Lipofectamine RNAiMAX (#13778-150, Invitrogen) for 48 h to achieve gene knockdown. Afterward, cells were stimulated with TNF-α for 24 h.

**mRNA expression level analysis by quantitative RT‑PCR**

Frozen atrial appendages or cell lysates were homogenized in 1 mL of QIAzol^R^ Qiagen (Hilden, Germany), RNA was extracted as per protocol recommendation (Qiagen). RNA was quantified using Nanodrop 1000 spectrophotometer machine (Thermo Fisher, Strasbourg, France) and a total of 1000 ng of RNA was reverse transcribed using Maxima first strand cDNA synthesis kit with dsDNase k1672 (Thermo Fisher) in My iQ 576BR0703 icycler (Biorad) to produce cDNA. Quantitative PCR was then performed using CFX connect 788BR2133 (Biorad) with a total of 10 ng of cDNA per well. The used primers are listed in the Supplemental Table 1. The mean of three different housekeeping genes were used for normalization and quantification was performed using the 2^-ΔΔCt formula.

**Western blot** **analysis**

Frozen atrial appendages were homogenized using liquid nitrogen, lysed in Ripa (Radioimmunoprecipitation assay) extraction buffer and sonicated. Following treatment, cells were washed with PBS at 4 °C and then lysed in Ripa buffer. Proteins were quantified according to Lowry protein assay. Thereafter, total proteins (10 and 20 μg for cells and tissues, respectively) were separated on denaturing SDS polyacrylamide gel, and then transferred onto nitrocellulose membranes. Blots were blocked with 5 % bovine serum albumin, then incubated with the respective primary antibody at 4 °C.

After washing with TBS-Tween 0.1 %, membranes were incubated with appropriated peroxidase-labeled secondary antibody (Cell Signaling Technology, Saint-Cyr L’École, France, cat. nº #7074, #7076). Clarity Western ECL substrate (BioRad, Hercules, California, USA) was used for detection of signals using the ChemiDoc imaging system (BioRad). Band optical density was quantified using ImageJ software and normalized to the corresponding housekeeping protein.

**Immunofluorescence staining**

Cryosections of 10 μm thickness were fixed with paraformaldehyde 4 % (w/v) for 30 min, washed thoroughly and then incubated with blocking/permeabilizing buffer (PBS containing 1 % BSA (w/v) and 0.5 % Triton X-100 (w/v)). After buffer removal, AECs and tissues were incubated with a primary antibody overnight at 4 °C. After washing with PBS, they were incubated with a 1:250 dilution of the appropriate secondary antibodies for 1 h at room temperature in the dark. After washing, tissues were incubated with 4’,6-diamidino-2’-phenylindole dihydrochloride (DAPI, Thermo Fisher) at room temperature, to counterstain nuclei. Afterwards, slides were mounted with a fluorescent mounting medium. Images were acquired using a Zeiss LSM 800 inverted confocal microscope.

**Colocalization analysis**

Colocalization analysis was performed on acquired RAA immunofluorescence images using ImageJ/Fiji software with the JaCoP (Just Another Colocalization Plugin). Images were background-subtracted prior to analysis. Regions of interest (ROIs) were defined at the single-cell level based on the staining of cell-type–specific markers, thereby restricting the analysis to the appropriate cell population. Pearson’s correlation coefficient was calculated between SGLT2 or GLP-1R and the corresponding cell-specific marker (Troponin T for cardiomyocytes, CD31 for endothelial cells, or CD86 for macrophages) within each ROI.

**Detection of ROS formation in RAA**

For *in situ* experiments, atrial appendage tissue cryosections (15 μm thickness) were either untreated and covered with the vehicle solution (PBS 1X) to prevent tissue dehydration or incubated with either N-acetylcysteine (NAC, an antioxidant, 1 mM for 2 h), VAS-2870 (NADPH oxidases inhibitor, 1 μM), empagliflozin (selective SGLT2 inhibitor, 100 nM), infliximab (TNF-α neutralizing antibody, 10 μg/mL), SQ22536 (adenylyl cyclase inhibitor, 50 μM), exendin 9-39 (GLP-1R antagonist, 200 nM), PKI (14-22) (selective protein kinase A inhibitor, 10 μM), semaglutide/liraglutide (GLP-1R agonists, 100 nM) or forskolin (adenylyl cyclase activator, 50 μM) for 30 min. Then, tissue sections were either untreated or exposed to TNF-α (10 ng/mL) for another 30 min. All used reagents were dissolved in vehicle for a final volume that uniformly covers all the tissue section (200 µL per section).

Thereafter, tissue sections were exposed to dihydroethidium (5 μM, a redox-sensitive fluorescent dye) for 30 min at 37 °C in the dark. After washing with PBS (Phosphate-buffered saline solution), samples were mounted with a fluorescent mounting medium (Fluoromount-G, 00-4958-02) from Invitrogen (Strasbourg, France). Images were acquired using a Zeiss LSM 800 inverted confocal microscope with a filter set for dihydroethidium (excitation at 518 nm and emission at 608 nm). Quantification was performed by ImageJ software where mean intensities were expressed as arbitrary densitometry units.

**Cellular Measurement of ROS formation**

Cells were exposed to the indicated treatments for 24 h. Intracellular ROS formation was then assessed by incubation with dihydroethidium (DHE; 5 μM) for 1 h at 37°C in 5% CO₂. After incubation, cells were washed once with PBS, and fluorescence intensity was measured using a Varioskan plate reader (Thermo Fisher Scientific) with excitation at 518 nm and emission at 608 nm (exposure time: 500 ms).

**Evaluation of collagen fibrosis distribution using Sirius red staining**

To evaluate fibrosis, paraffin sections were cut at 4 µm, dewaxed, and hydrated, then stained according to the manufacturer’s instructions using the Sirius Red Coloration Kit DK14145 (LABOMODERNE, Paris, France). Sections were observed using bright field microscope (AMG, EVOS XL CORE). Quantification was performed by ImageJ software where fibrosis was expressed as area percentage of collagen fibrosis distribution.

**Supplemental Table 1. Inverse propability of treatment weighting adjusted analysis.**

| **Non-adjusted cohort** | | | | |
| --- | --- | --- | --- | --- |
| ***Clinical factors*** | **SR (n=61)** | **AF (n=17)** | ***p* value** | **SMD** |
| **Age (years)** | 65.0 [55.0- 71.0] | 75.0[62.0- 78.0] | 0.01 | 0.75 |
| **Sex (Male)** | 47 (77.0) | 15 (88.2) | 0.50 | 0.30 |
| **Body Mass Index (kg/m^2^)** | 26.8 [23.3- 29.9] | 25.20 [23.3- 28] | 0.30 | 0.17 |
| **eGFR <60 mL/min/1.73 m^2^** | 5 (8.6) | 2 (12.5) | >0.99 | 0.13 |
| **Diabetes** | 10 (16.4) | 3 (17.6) | >0.99 | 0.03 |
| **Dyslipidemia** | 30 (49.2) | 9 (52.9) | >0.99 | 0.08 |
| **Hypertension** | 33 (54.1) | 12 (70.6) | 0.35 | 0.35 |
| **LVEF<60 %** | 11 (18.0) | 6 (35.3) | 0.23 | 0.40 |
| **Stroke** | 0 (0.0) | 1 (5.9) | 0.49 | 0.35 |
| **Vascular pathology** | 14 (23.0) | 5 (29.4) | 0.82 | 0.14 |
| ***Markers*** | | | | |
| **IL1B** | 0.32 ± 0.06 | 3.33 ± 0.83 | <0.001 | 1.23 |
| **IL6** | 0.55 ± 0.08 | 4.54 ± 0.87 | <0.001 | 1.56 |
| **TNFA** | 0.82 ± 0.09 | 4.95 ± 0.84 | <0.001 | 1.65 |
| **CD68** | 0.45 ± 0.10 | 4.43 ± 0.82 | <0.001 | 1.61 |
| **CCL2** | 1.01 ± 0.14 | 4.35 ± 0.92 | <0.001 | 1.20 |
| **SLC5A1** | 0.66 ± 0.11 | 4.99 ± 0.84 | <0.001 | 1.71 |
| **SLC5A2** | 0.38 ± 0.11 | 5.02 ± 0.96 | <0.001 | 1.63 |
| **GLP1R** | 1.48 ± 0.09 | 4.72 ± 0.75 | <0.001 | 1.45 |
| **NOS3** | 2.00 ± 0.27 | 0.22 ± 0.05 | 0.001 | 1.19 |
| **IPTW-adjusted cohort** | | | | |
| ***Clinical factors*** | **SR (n=61)** | **AF (n=17)** | ***p* value** | **SMD** |
| **Age (years)** | 66.0 [26.0- 80.0] | 62.1 [52.0- 84.0] | 0.70 | 0.24 |
| **Sex (Male)** | 47.6 (79.1) | 13.6 (87.4) | 0.47 | 0.22 |
| **Body Mass Index (kg/m^2^)** | 26.5 [20.4- 37.0] | 25.5 [18.8-40.9] | 0.99 | 0.18 |
| **eGFR <60 mL/min/1.73 m^2^** | 5.8 (10.2) | 2.0 (15.5) | 0.60 | 0.16 |
| **Diabetes** | 9.4 (15.6) | 1.9 (12.0) | 0.70 | 0.11 |
| **Dyslipidemia** | 28.8 (47.9) | 7.9 (50.3) | 0.89 | 0.05 |
| **Hypertension** | 34.5 (57.4) | 10.3 (65.8) | 0.61 | 0.17 |
| **LVEF<60 %** | 13.5 (22.5) | 3.6 (23.1) | 0.96 | 0.02 |
| **Stroke** | 0.0 (0.0) | 0.2 (1.4) | 0.07 | 0.17 |
| **Vascular pathology** | 13.9 (23.1) | 2.2 (14.3) | 0.38 | 0.23 |
| ***Markers*** | | | | |
| **IL1B** | 0.31 ± 0.06 | 2.58 ± 0.78 | 0.003 | 1.03 |
| **IL6** | 0.54 ± 0.07 | 3.90 ± 0.84 | <0.001 | 1.42 |
| **TNFA** | 0.81 ± 0.08 | 4.37 ± 0.82 | <0.001 | 1.53 |
| **CD68** | 0.43 ± 0.10 | 3.99 ± 0.79 | <0.001 | 1.56 |
| **CCL2** | 1.04 ± 0.14 | 4.74 ± 1.07 | 0.008 | 1.20 |
| **SLC5A1** | 0.64 ± 0.11 | 5.09 ± 0.88 | <0.001 | 1.76 |
| **SLC5A2** | 0.36 ± 0.10 | 5.17 ± 1.04 | <0.001 | 1.63 |
| **GLP1R** | 1.48 ± 0.09 | 5.21 ± 0.88 | 0.001 | 1.49 |
| **NOS3** | 1.95 ± 0.26 | 0.20 ± 0.05 | <0.001 | 1.24 |

Data of clinical factors are presented as count (%) or median [25th-75th percentiles]. Data of markers are shown as mean ± standard error. SMD < 0.25 was deemed acceptable due to the small sample size suggesting covariate balance between groups groups^2^.

IPTW: inverse propability of treatment weighting; SMD: standardized mean difference; SR: Sinus rhythm; AF: Atrial fibrillation; LVEF: Left ventricular ejection fraction; eGFR: estimated glomerular filtration rate. CCL2, monocyte chemoattractant protein-1 (MCP-1); CD68, cluster of differentiation 68 (CD68); GLP1R, glucagon-like peptide-1 receptor (GLP-1R); IL1B, interleukin-1β (IL-1β); IL6, interleukin-6 (IL-6); NOS3, endothelial nitric oxide synthase (eNOS); SLC5A1, sodium–glucose cotransporter 1 (SGLT1); SLC5A2, sodium–glucose cotransporter 2 (SGLT2); TNFA, tumor necrosis factor-α (TNF-α).

**Supplemental Table 2. Primer sequences for real-time quantitative PCR analysis and siRNA transfection**

| ***Gene*** | **Protein** | **Forward Sequence** | **Reverse Sequence** |
| --- | --- | --- | --- |
| *ACTB* | **β-Actin** | GCCAGGGCTTACCTGTACACT | CATTTTTAAGGTGTGCACTTTTATTC |
| *CCL2* | **MCP1** | CACCTTCATTCCCCAAGGGC | ACACTTGCTGCTGGTGATTCT |
| *CD163* | **CD163** | TGTGGTCTTACTTCTCAGTGCC | GCCTCAGCTCCTTGTCTGTT |
| *CD68* | **CD68** | TCTTTCACCAGCTGTCCACC | CACTGGGGCAGGAGAAACTT |
| *CD86* | **CD86** | ACCAACACAATGGAGAGGGAA | AAAACACGCTGGGCTTCATC |
| *CDKN1A* | **p21** | GGCAGACCAGCATGACAGATT | AGGGCTTCCTCTTGGAGAAGAT |
| *CDKN2A* | **p16** | CGCGATGTCGCACGGTA | TCTATGCGGGCATGGTTACT |
| *COL3A1* | **Collagen-3** | ACACAGAGGCTTCGATGGAC | GAGCCCCTCTTGGACCCAT |
| *F3* | **TF** | GGGAACCCAAACCCGTCAAT | GTCGGTGAGGTCACACTCTG |
| *GAPDH* | **GAPDH** | AGCCACATCGCTCAGACAC | GCCCAATACGACCAAATCC |
| *GLP1R* | **GLP-1R** | GAGAATACCGACGCCAGTGC | AGGTCCGGTTGCAGAACAAG |
| *GUSB* | **GUSB** | CGCCCTGCCTATCTGTATTC | TCCCCACAGGGAGTGTGTAG |
| *IL1B* | **IL-1β** | GCAGAAGTACCTGAGCTCGC | AAGTCATCCTCATTGCCACTGT |
| IL6 | **IL-6** | CCACCGGGAACGAAAGAGAA | GAGAAGGCAACTGGACCGAA |
| *MMP9* | **MMP-9** | AGAACCAATCTCACCGACAGG | TTTCGACTCTCCACGCATCTC |
| *NOS3* | **eNOS** | GACCCTCACCGCTACAACAT | CCGGGTATCCAGGTCCAT |
| *SLC5A1* | **SGLT1** | GTTTGCTTATGGAACCGGGAG | TGGCGAAGAGGATAATGGCA |
| *SLC5A2* | **SGLT2** | ATCTATGCCTCCGTCATCGC | GAAGGTCTGTACCGTGTCCG |
| *TGFB1* | **TGF-β1** | GCCCTCGGGAGTCGC | CCTGGAGGAGAAAGGGTCTAGG |
| TNFA | **TNF-α** | CTGCACTTTGGAGTGATCGG | CTCGGGGTTCGAGAAGATGA |
| *TP53* | **p53** | AGTCACAGCACATGACGGAG | ACCATCGCTATCTGAGCAGC |

CD: Cluster of differentiation; eNOS: Endothelial nitric oxide synthase; GAPDH: Glyceraldehyde-3-phosphate dehydrogenase; GLP-1R: glucagon-like peptide-1 receptor; GUSB: Glucuronidase beta; IL-1β: Interleukin-1 beta; IL-6: Interleukin-6; MCP-1: Monocyte chemoattractant protein-1; MMP-9: matrix metallopeptidase 9;p16: Cyclin-dependent kinase inhibitor 2A; p21: Cyclin-dependent kinase inhibitor 1A; p53: Tumor protein P53; SGLT: Sodium-glucose co-transporter; TF: Tissue factor; TGF-β1: Transforming growth factor beta-1; TNF-α: Tumor necrosis factor-alpha.

**Supplemental Figures**

**Supplemental Figure 1. Protein expression and fibrosis in RAA**.

**
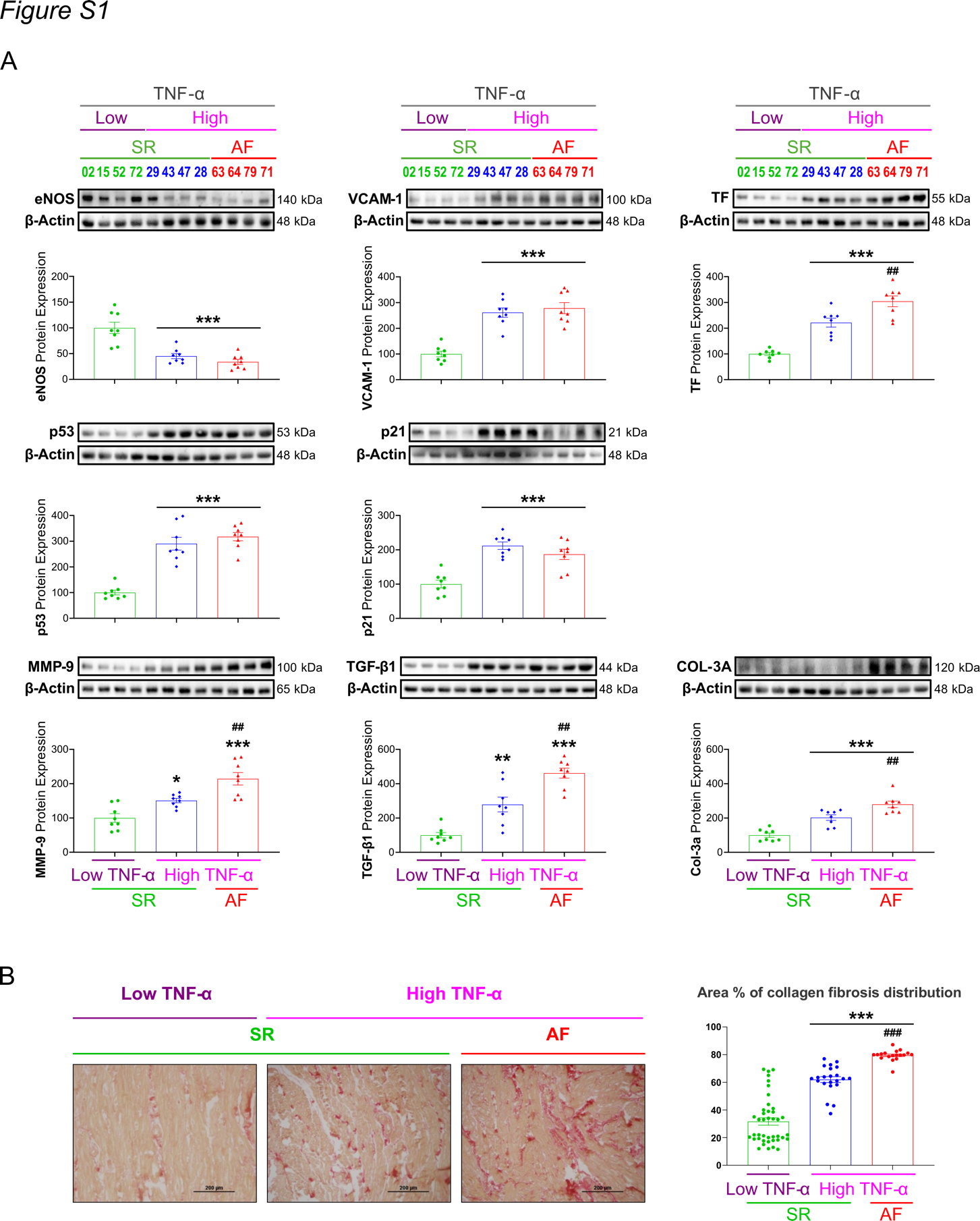
**

Three groups of right atrial appendage (RAA) samples were analyzed according to TNF-α gene expression levels: (1) samples with low TNF-α expression (below median), (2) samples with high TNF-α expression in patients with sinus rhythm (SR; above median), and (3) samples with high TNF-α expression in patients with a history of atrial fibrillation (AF). Protein expression of selected markers was assessed by Western blot analysis and is presented as representative immunoblots (n = 4 patients per group) together with corresponding cumulative data from eight patients per group (A). The percentage area of collagen fibrosis distribution among the three groups was assessed by Sirius red staining, shown as representative images and cumulative data (B). Data are expressed as mean ± SEM. Statistical significance was determined by one-way ANOVA followed by Tukey’s test : **P* < 0.05 versus low TNF-α, *^#^P* < 0.05 versus high TNF-α SR. Significance levels are indicated as **P* < 0.05, ***P* < 0.01, ****P* < 0.001, *^##^P* < 0.01and *^###^P* < 0.001.


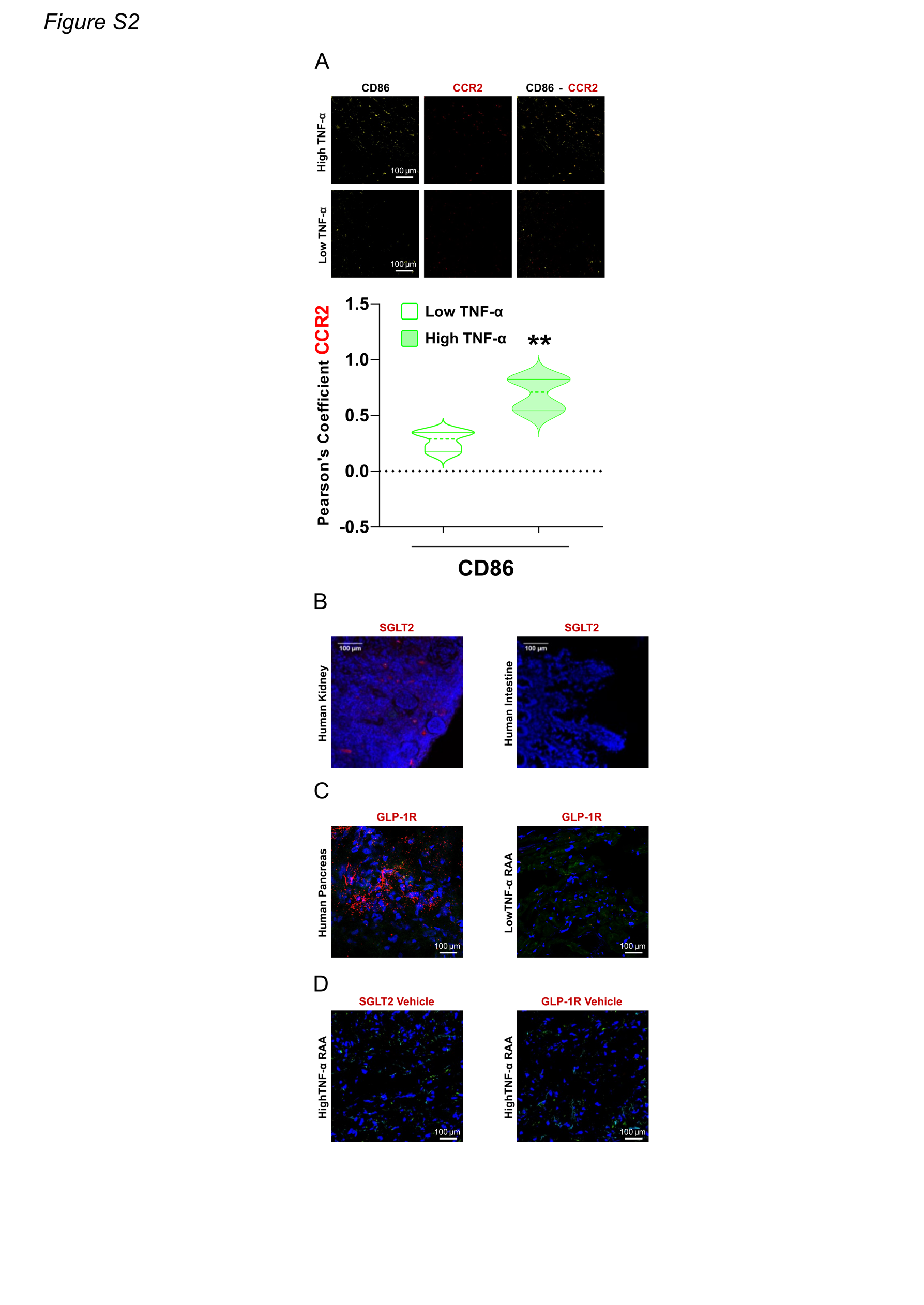

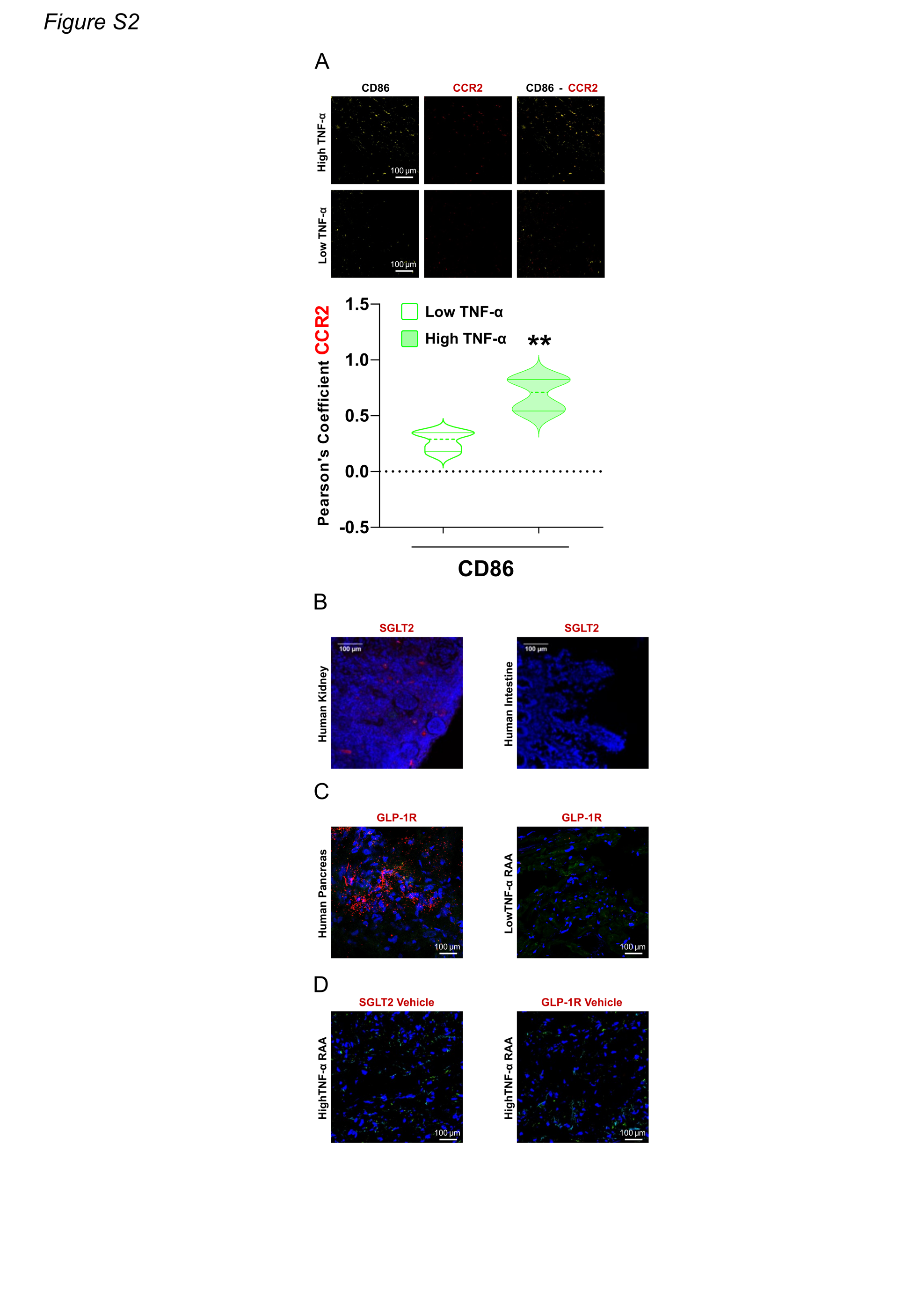
**Supplemental Figure 2. Validation of immunofluorescence specificity.**

Right atrial appendage (RAA) tissue sections with high or low TNF-α expression were stained for CD86 and CCR2 (A). Colocalization was quantified using the Pearson correlation coefficient, comparing high versus low TNF-α expression groups. Comparisons between low and high TNF-α groups were performed using an unpaired t-test (***P* < 0.01 vs low TNF-α RAA) (A).

Reference human tissues were used as positive and negative controls to validate antibody specificity. For SGLT2, immunofluorescence staining was performed on human kidney tissue (positive control) and human intestinal tissue with low SGLT2 expression (negative control), as previously reported^3^. GLP-1R staining was performed on human pancreas tissue as a positive control and on RAA sections from the low TNF-α group as a negative control. Sections processed in the absence of primary antibody were also used as negative controls. Human tissue samples were provided by the Centre de Ressources Biologiques, University Hospital of Strasbourg, France. Nuclei were counterstained with DAPI (blue) (B).


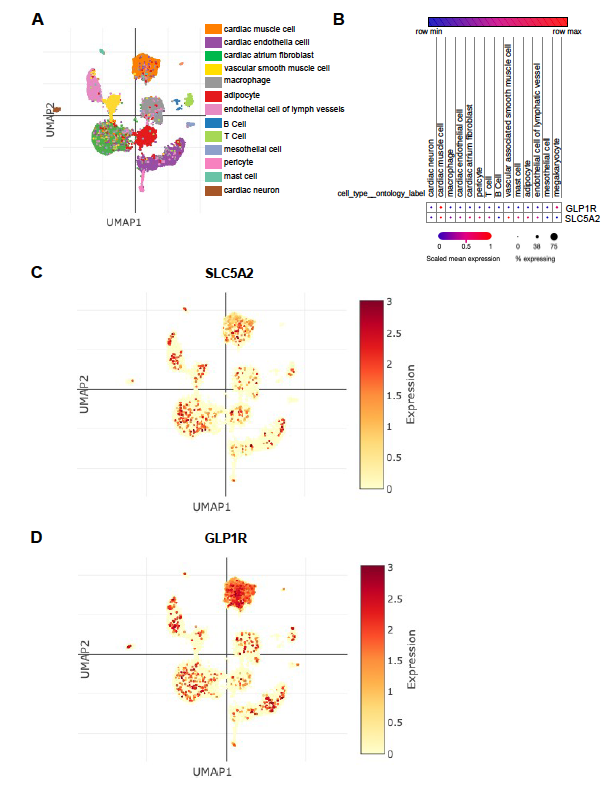
**Supplemental Figure 3. Cell-type–specific expression of SGLT2 and GLP-1R in the human atrium**

UMAP embedding of atrial nuclei from left atrial tissue of patients with atrial fibrillation and control individuals, colored by annotated cell type; identities are indicated in the legend (A). Dot plot showing SLC5A2 and GLP1R expression across cell types; dot size indicates the percentage of nuclei expressing each gene, and color indicates average expression (B). UMAP highlighting SLC5A2 (C) and GLP1R (D) expression across cell types, with expression intensity indicated by the color scale.

**Supplemental Figure 4.** **Validation of SGLT2 and GLP-1R Knockdown in Human Cells.**


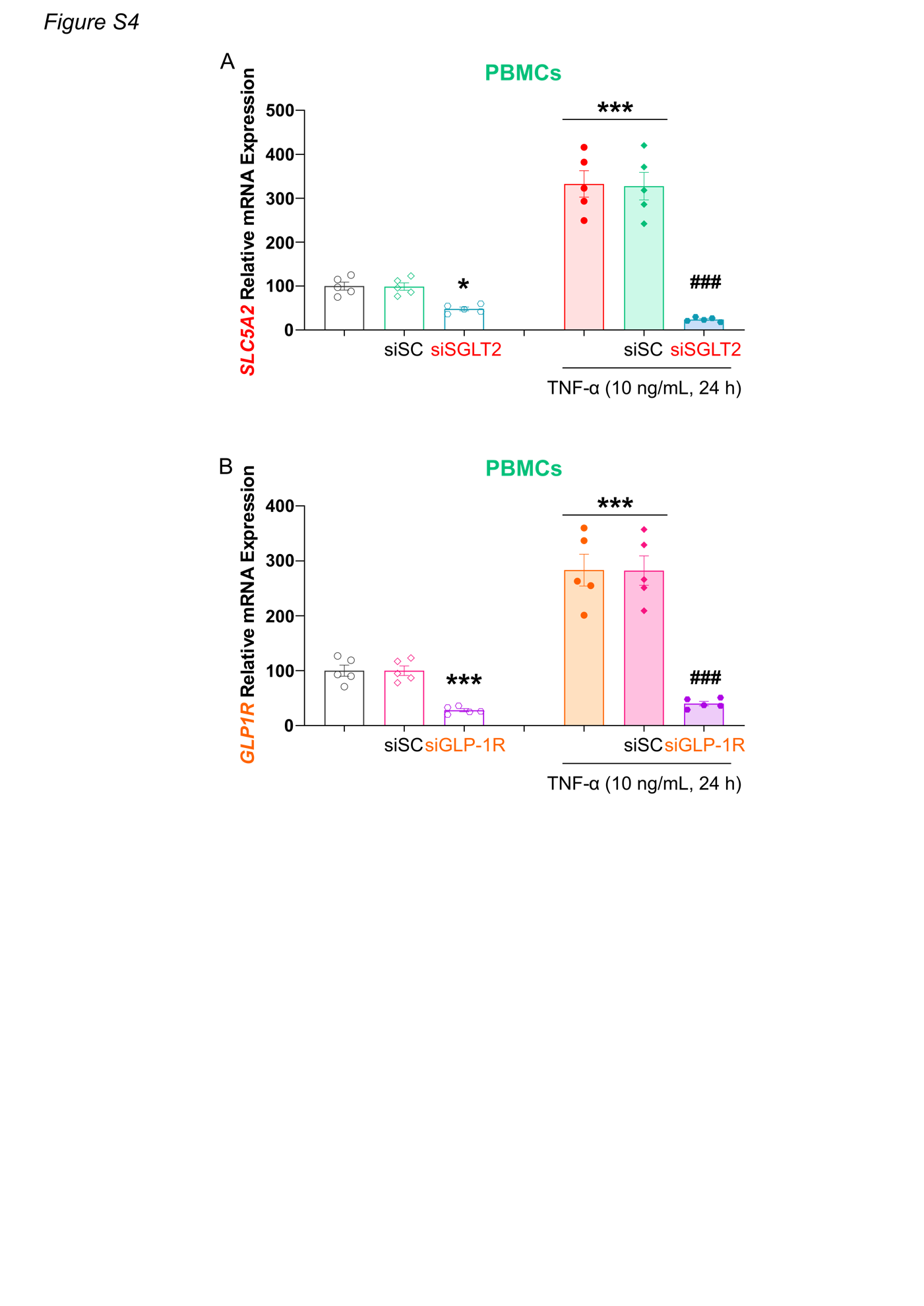
Expression levels of SGLT2 (A) and GLP-1R (B) were assessed in human peripheral blood mononuclear cells (PBMCs) following 48 h of gene knockdown using specific siRNAs. Scrambled siRNA (Scrbl) served as the negative control. Cells were subsequently stimulated with TNF-α (10 ng/mL) for 24 h, and mRNA levels were analyzed by RT-qPCR. Data are presented as mean ± SEM from n = 5 independent experiments. **P < 0.05* vs. Control ; *#P < 0.05* vs. TNF-α-stimulated cells, analyzed by one-way ANOVA followed by Tukey’s multiple comparison test. Significance levels are indicated as **P* < 0.05, ****P* < 0.001 and *^###^P* < 0.001.


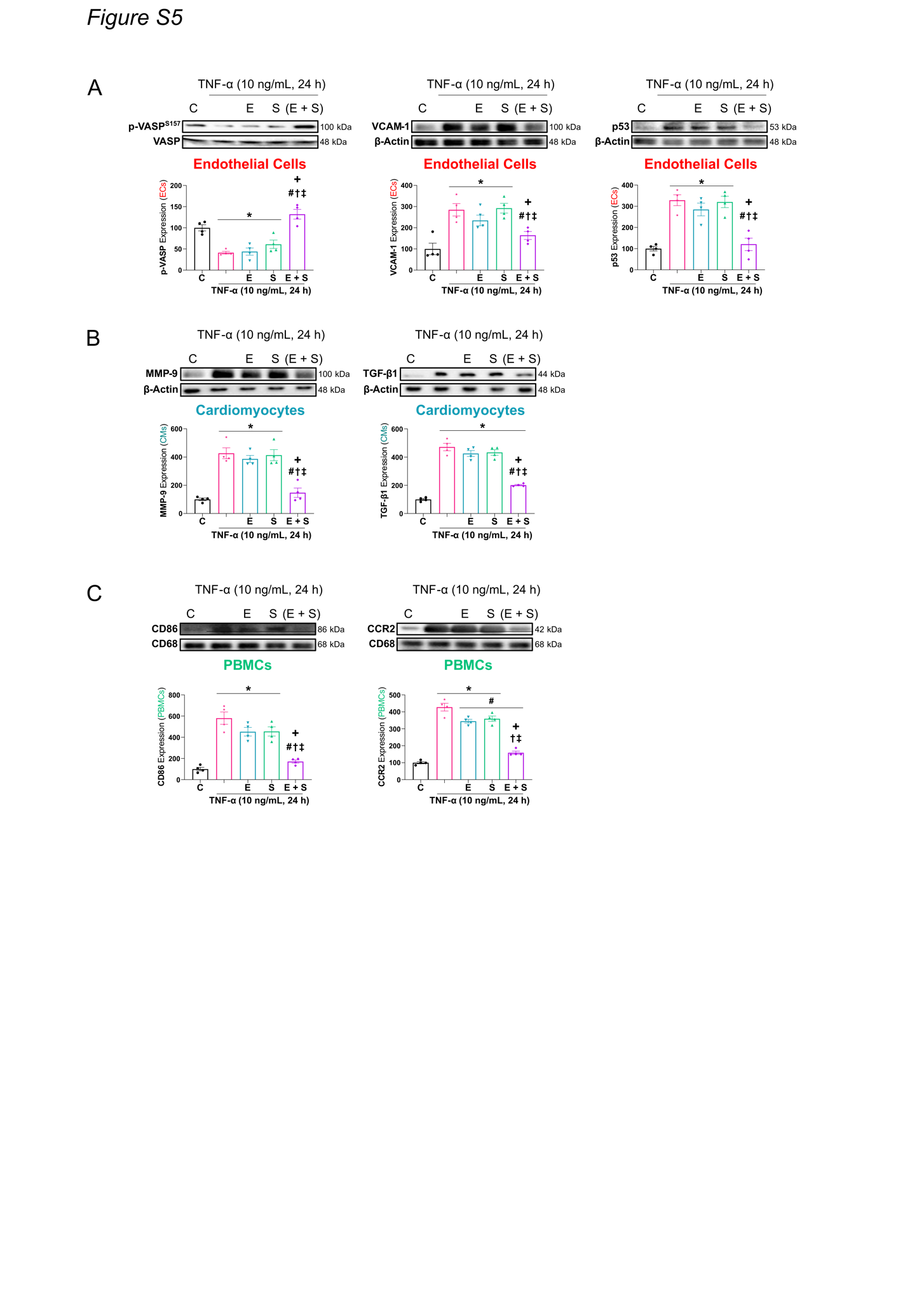
**Supplemental Figure 5.** **Antioxidant and anti-inflammatory effects of SGLT2i plus GLP-1Ra.**

Human coronary artery endothelial cells (hCAECs, ECs; C), human cardiomyocytes (AC16, CMs; D), and human peripheral blood mononuclear cells (PBMCs; E) were pretreated with empagliflozin (EMPA, 10 nM), semaglutide (SEMA, 10 nM), or their combination (E+S) for 30 min prior to TNF-α stimulation (10 ng/mL) for 24 h, followed by assessment of target protein expression by Western blot. Data shown in arbitrary units (AU) as mean ± SEM. **P* < 0.05 versus control, ^#^*P* < 0.05 versus TNF-α, ^†^*P* < 0.05 versus empagliflozin, ^‡^*P* < 0.05 versus semaglutide and ^+^*P* < 0.05 versus additive effects of empagliflozin plus semaglutide analyzed using one-way ANOVA followed by Tukey’s multiple comparison test.

**References:**

1. Hill MC, Simonson B, Roselli C, et al. Large-scale single-nuclei profiling identifies a role for ATRNL1 in atrial fibrillation. *Nat Commun*. 2024;15:10002.
2. Jackson JW, Schmid I, Stuart EA. Propensity scores in pharmacoepidemiology: beyond the horizon. *Curr Epidemiol Rep*. 2017;4:271–280.
3. Bruckert C, Matsushita K, Mroueh A, et al. Empagliflozin prevents angiotensin II–induced hypertension-related micro- and macrovascular endothelial cell activation and diastolic dysfunction in rats despite persistent hypertension: role of endothelial SGLT1 and SGLT2. *Vasc Pharmacol*. 2022;146:107095.
