# Supplementary material for: Synergistic Effects of SGLT2 Inhibitors and GLP-1R Agonists on Inflammation-Associated Oxidative Stress in Atrial Fibrillation
Source: JACC Basic Transl Sci. 2026 Jun 6;11(7):101587. doi: 10.1016/j.jacbts.2026.101587 (PMC13266112; doi:10.1016/j.jacbts.2026.101587)
Supplement: Supplemental Material 2 [file mmc2.pdf]

Original Blots

Figure 2A

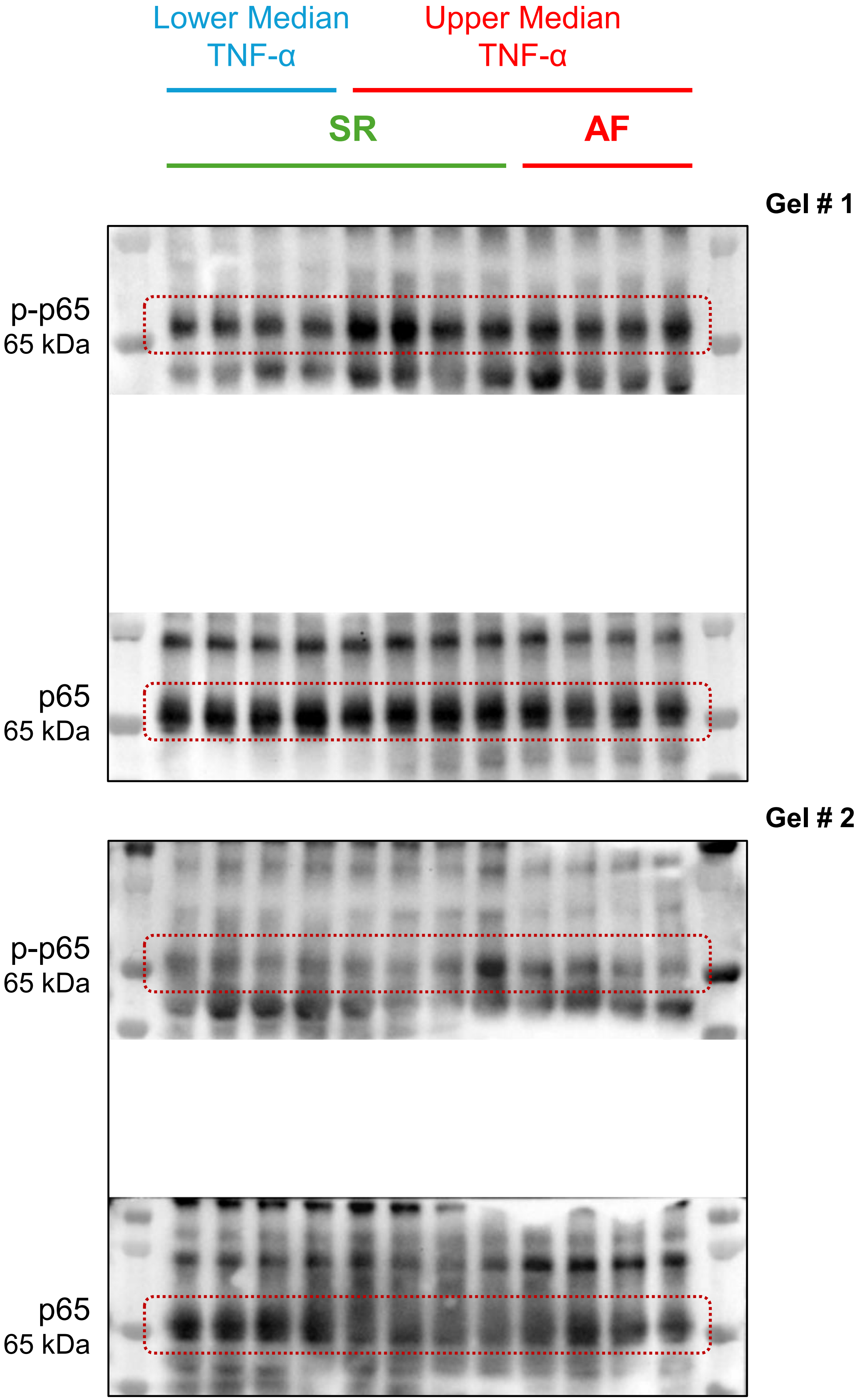
Lower Median
TNF-α
Upper Median
TNF-α
SR
AF
Gel # 1
p-p65
65 kDa
p65
65 kDa
Gel # 2
p-p65
65 kDa
p65
65 kDa

Figure 2A

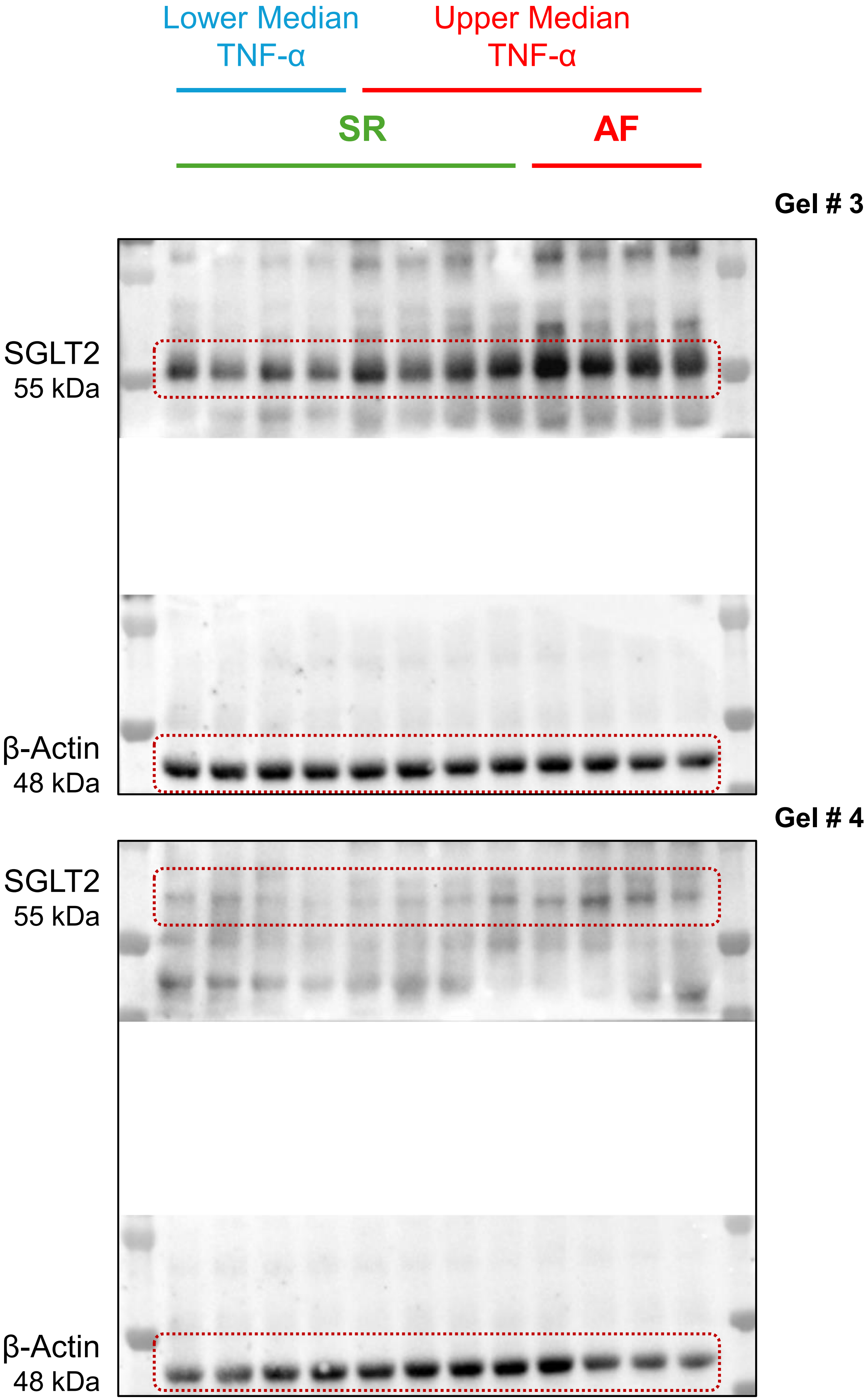

Lower Median
TNF-α
Upper Median
TNF-α
SR
AF
Gel # 3
SGLT2
55 kDa
β-Actin
48 kDa
Gel # 4
SGLT2
55 kDa
β-Actin
48 kDa

Figure 2A

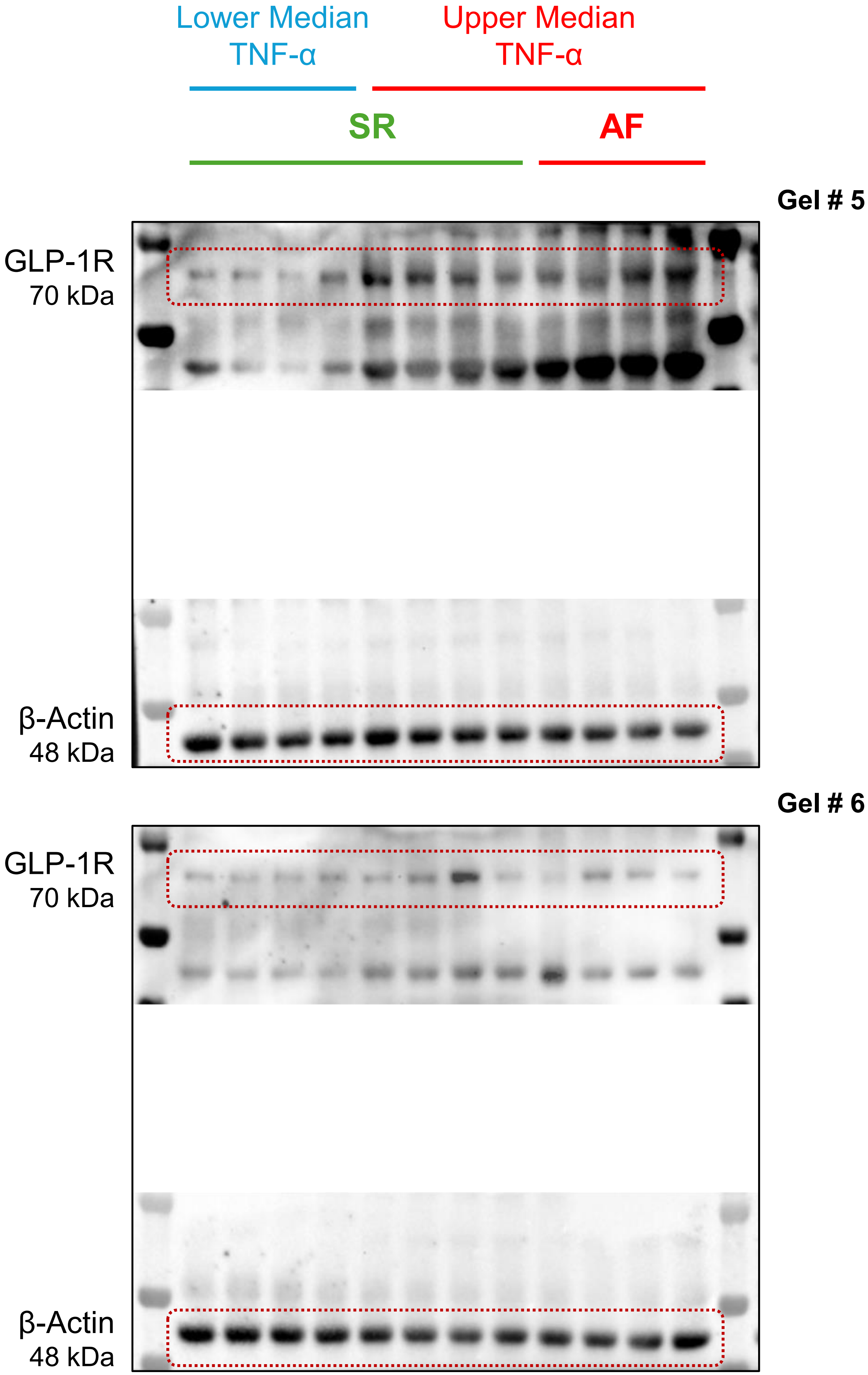
Lower Median TNF-α
Upper Median TNF-α
SR
AF
Gel # 5
GLP-1R
70 kDa
β-Actin
48 kDa
Gel # 6
GLP-1R
70 kDa
β-Actin
48 kDa

Figure 2A

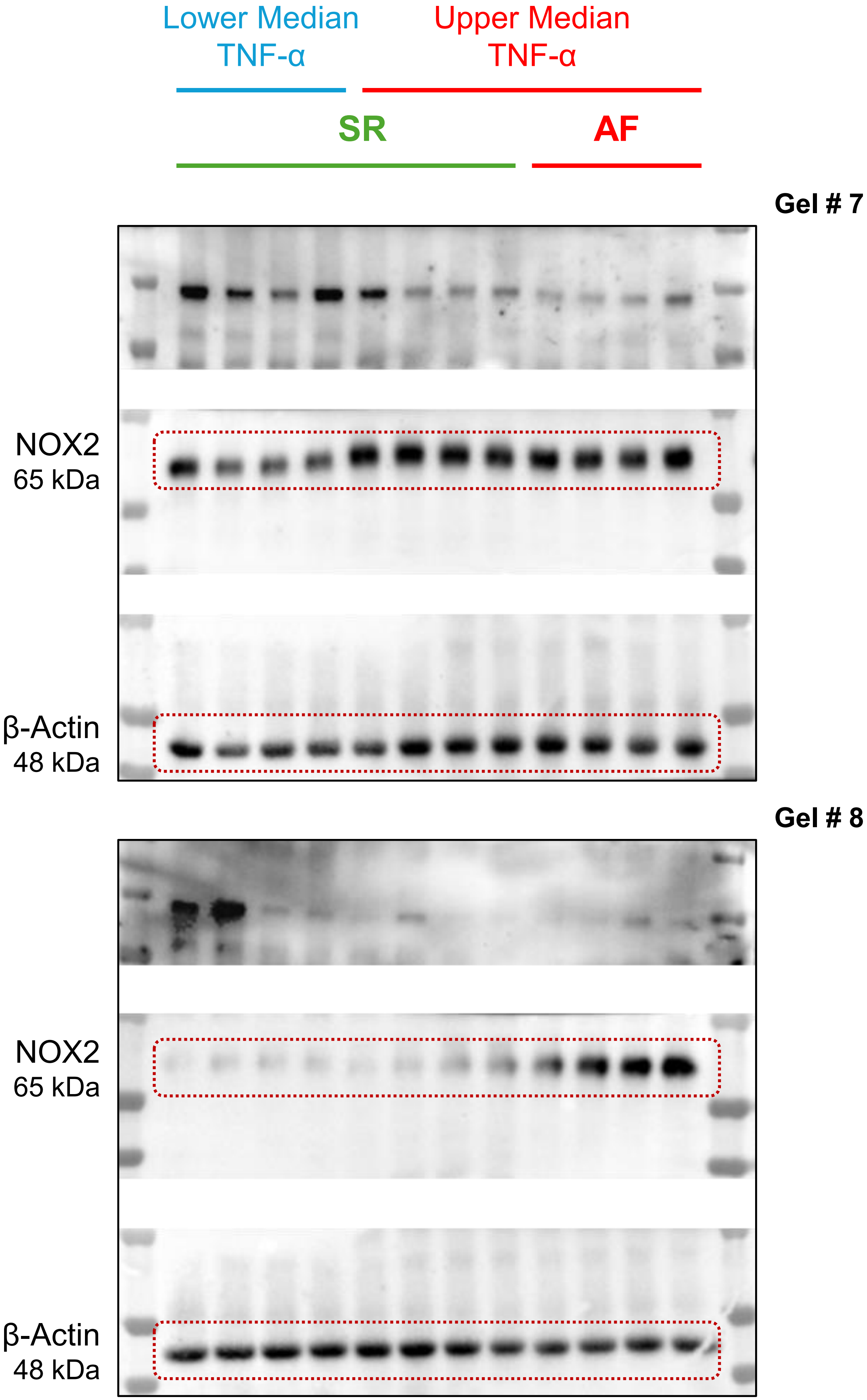
Lower Median
TNF-α
Upper Median
TNF-α
SR
AF
Gel # 7
NOX2
65 kDa
β-Actin
48 kDa
Gel # 8
NOX2
65 kDa
β-Actin
48 kDa

Figure 2C

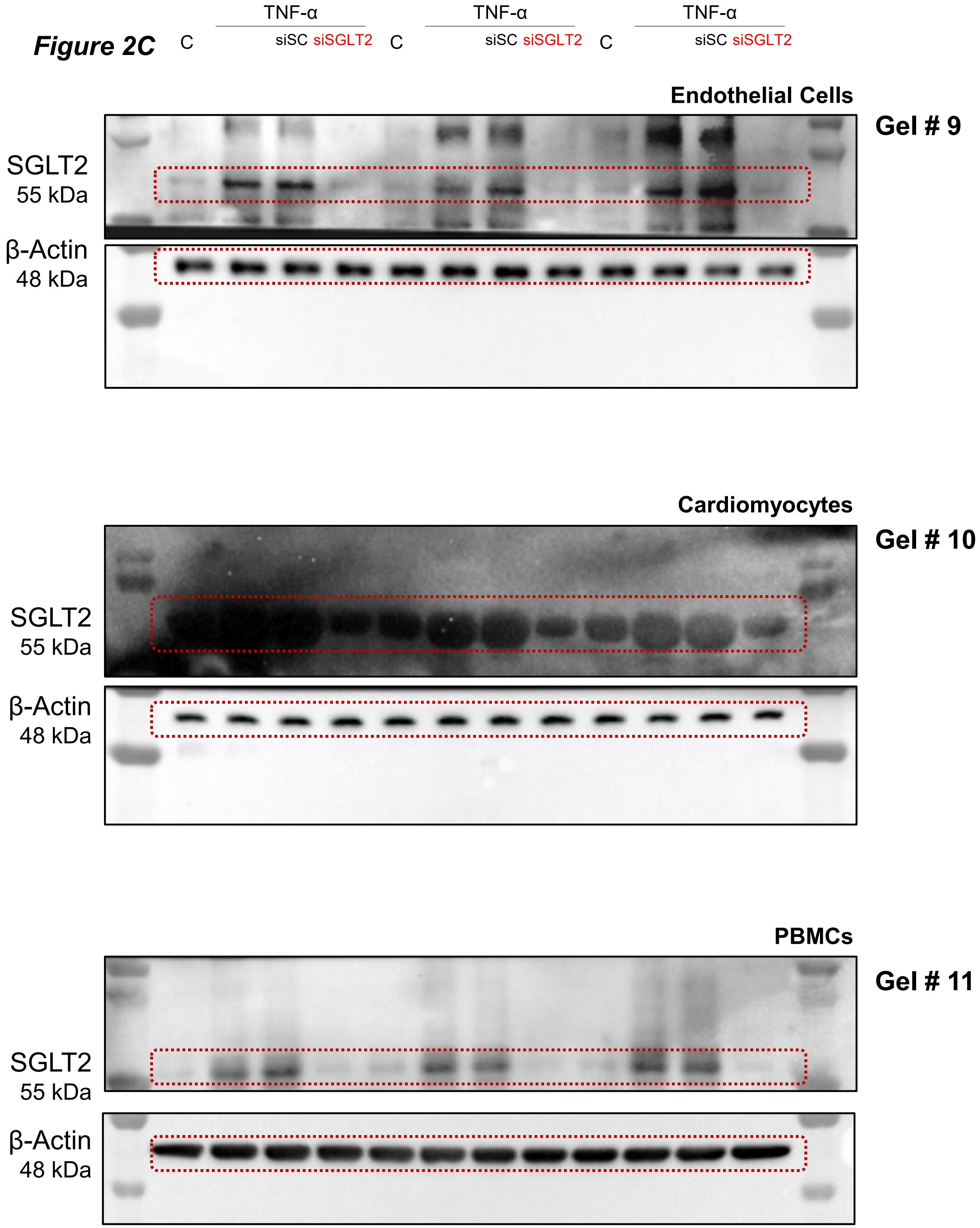

TNF-α
TNF-α
TNF-α
C
siSC
siSGLT2
C
siSC
siSGLT2
C
siSC
siSGLT2
Endothelial Cells
Gel # 9
SGLT2
55 kDa
β-Actin
48 kDa
Cardiomyocytes
Gel # 10
SGLT2
55 kDa
β-Actin
48 kDa
PBMCs
Gel # 11
SGLT2
55 kDa
β-Actin
48 kDa

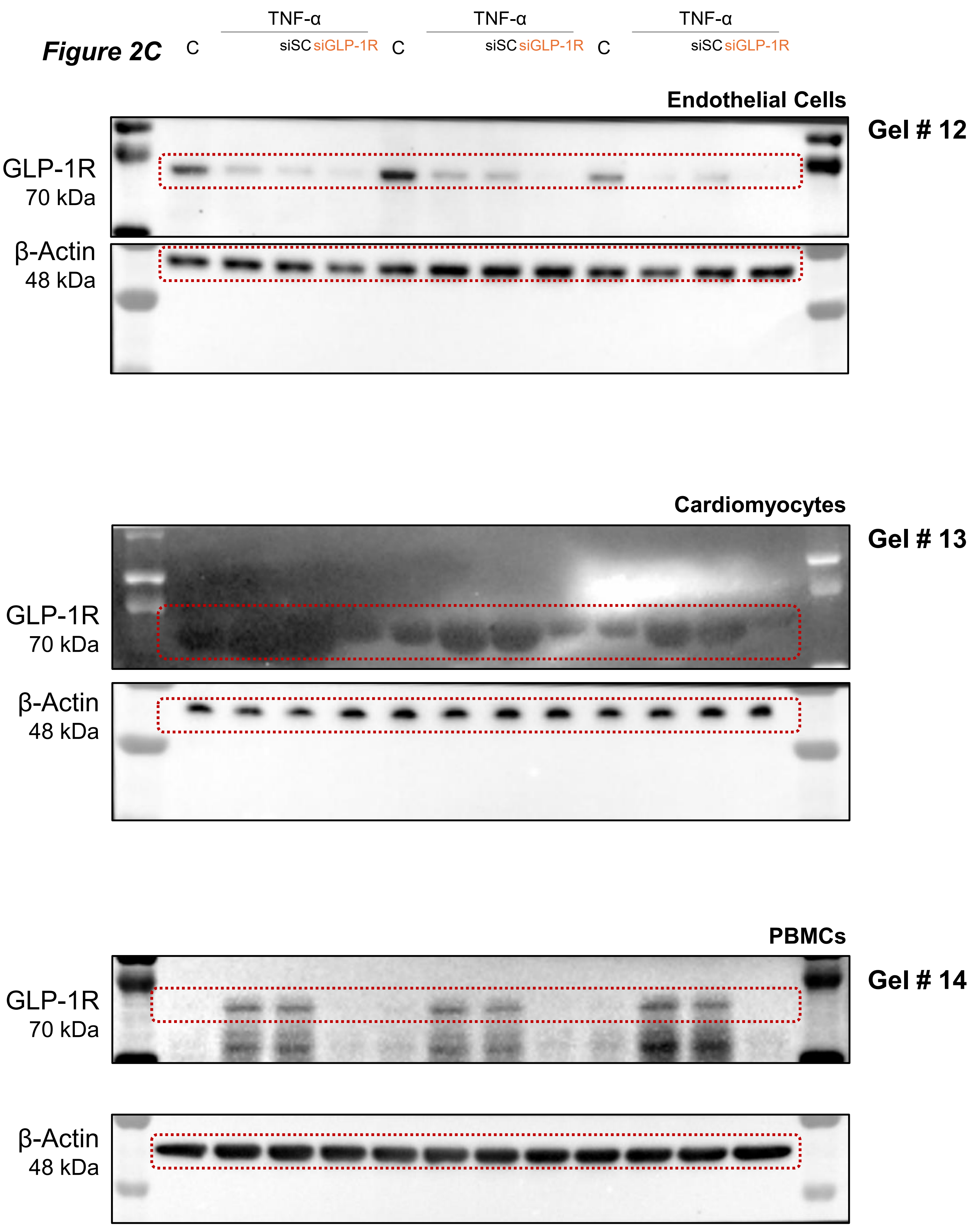
Figure 2C
C
TNF-α
siSC siGLP-1R
C
TNF-α
siSC siGLP-1R
C
TNF-α
siSC siGLP-1R
Endothelial Cells
Gel # 12
GLP-1R
70 kDa
β-Actin
48 kDa
Cardiomyocytes
Gel # 13
GLP-1R
70 kDa
β-Actin
48 kDa
PBMCs
Gel # 14
GLP-1R
70 kDa
β-Actin
48 kDa

Figure 5D

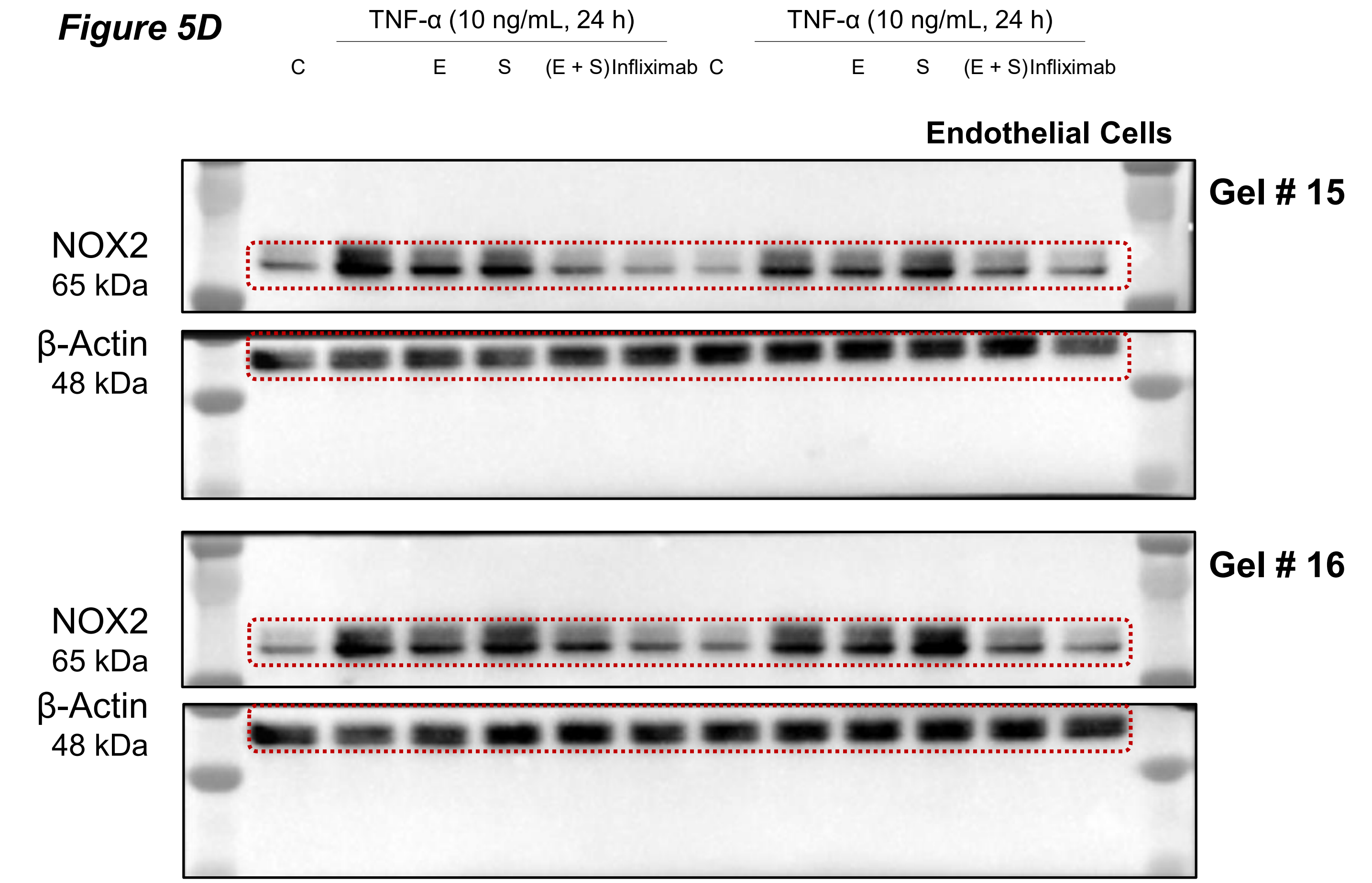
TNF-α (10 ng/mL, 24 h)
TNF-α (10 ng/mL, 24 h)
C E S (E + S) Infliximab C E S (E + S) Infliximab
Endothelial Cells
NOX2
65 kDa
β-Actin
48 kDa
Gel # 15
NOX2
65 kDa
β-Actin
48 kDa
Gel # 16

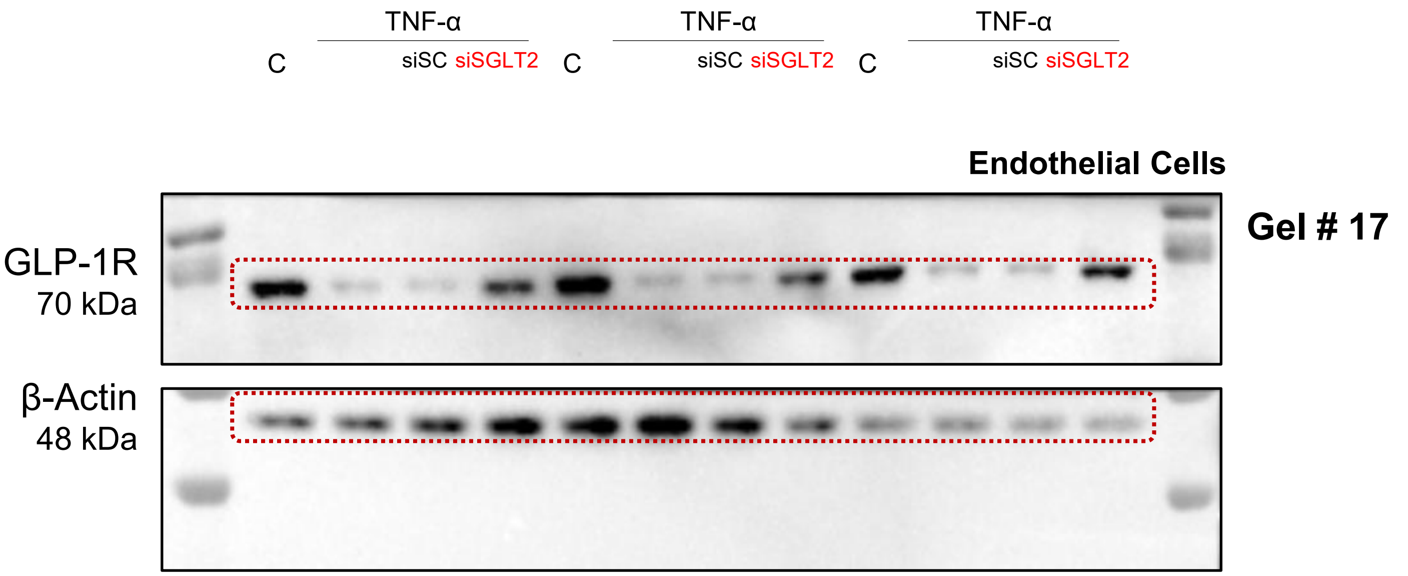
C
TNF-α
siSC siSGLT2
C
TNF-α
siSC siSGLT2
C
TNF-α
siSC siSGLT2
Endothelial Cells
GLP-1R
70 kDa
β-Actin
48 kDa
Gel # 17

Figure 5D

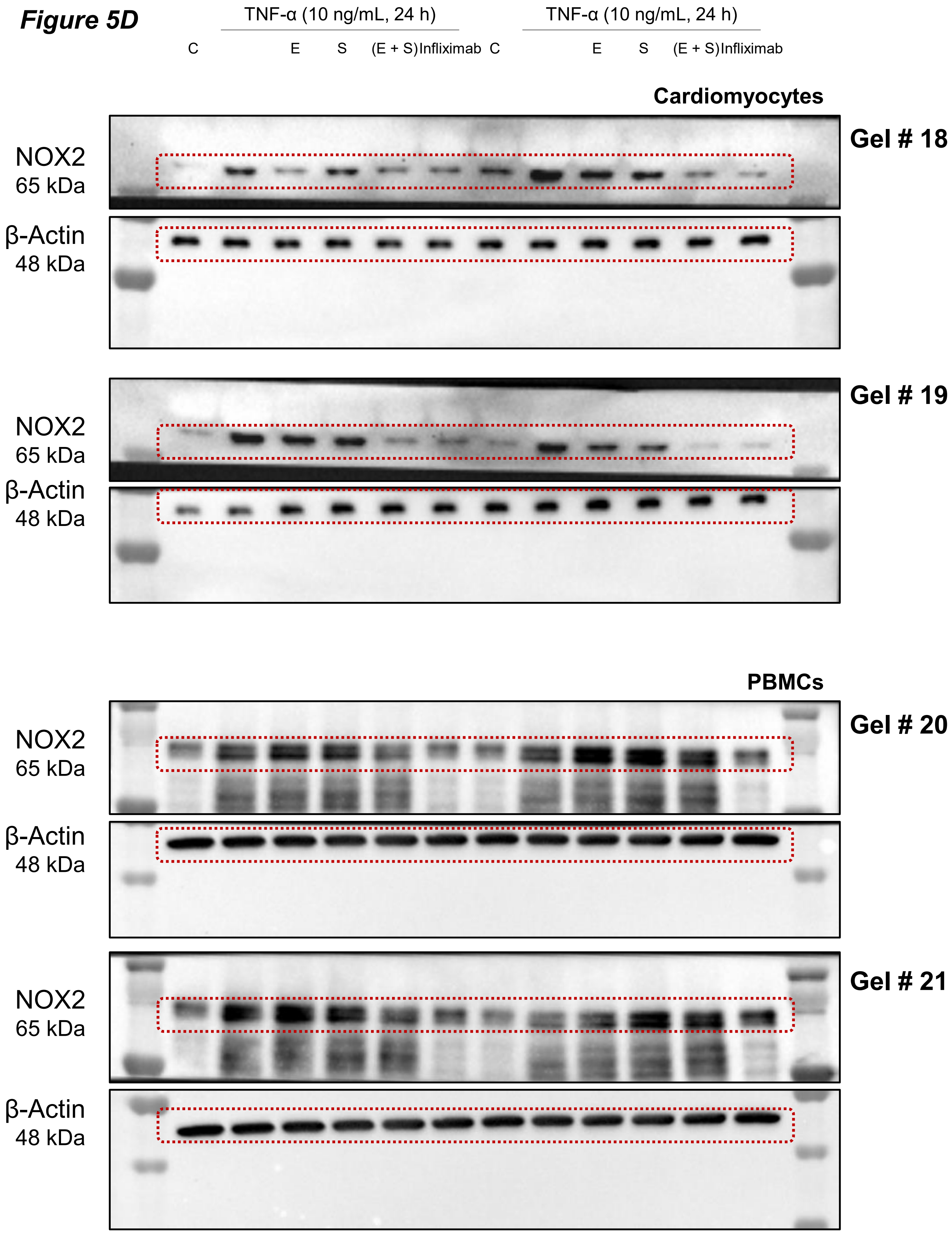

TNF-α (10 ng/mL, 24 h)
TNF-α (10 ng/mL, 24 h)
C
E
S
(E + S)
Infliximab
C
E
S
(E + S)
Infliximab
Cardiomyocytes
NOX2
65 kDa
β-Actin
48 kDa
Gel # 18
NOX2
65 kDa
β-Actin
48 kDa
Gel # 19
PBMCs
NOX2
65 kDa
β-Actin
48 kDa
Gel # 20
NOX2
65 kDa
β-Actin
48 kDa
Gel # 21

Figure S1A

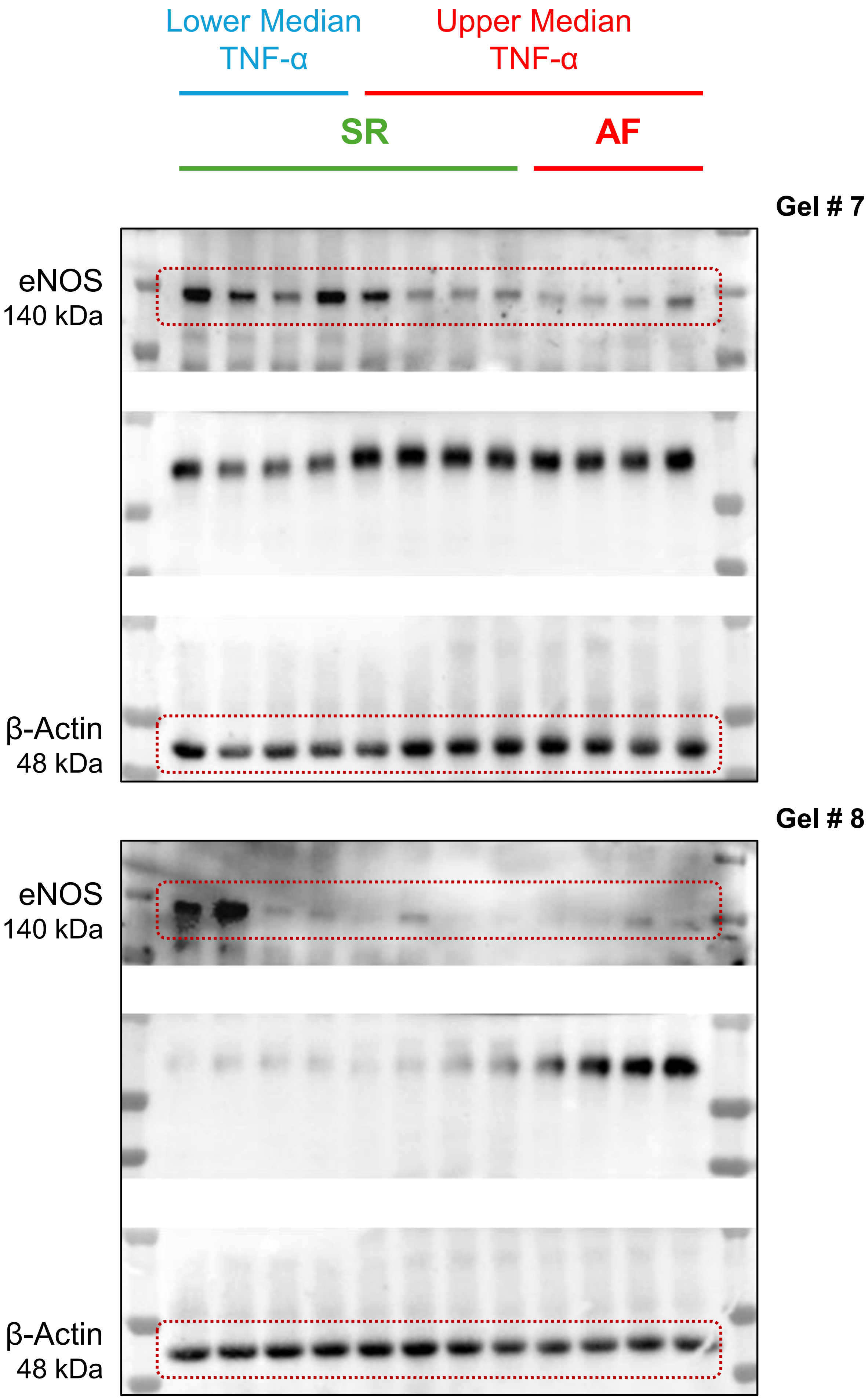
Lower Median
TNF-α
Upper Median
TNF-α
SR
AF
Gel # 7
eNOS
140 kDa
β-Actin
48 kDa
Gel # 8
eNOS
140 kDa
β-Actin
48 kDa

Figure S1A

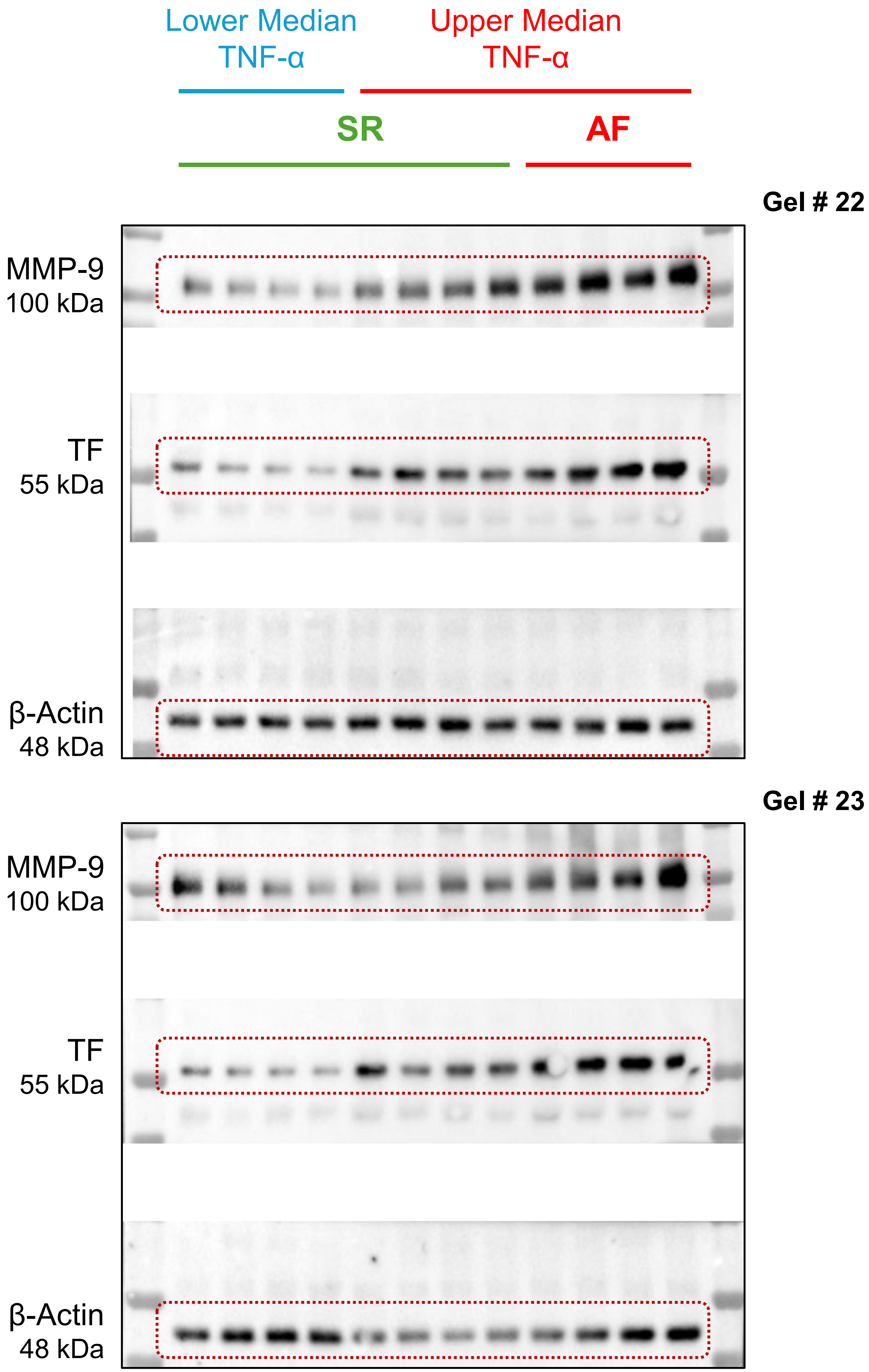
Lower Median
TNF-α
Upper Median
TNF-α
SR
AF
Gel # 22
MMP-9
100 kDa
TF
55 kDa
β-Actin
48 kDa
Gel # 23
MMP-9
100 kDa
TF
55 kDa
β-Actin
48 kDa

Figure S1A

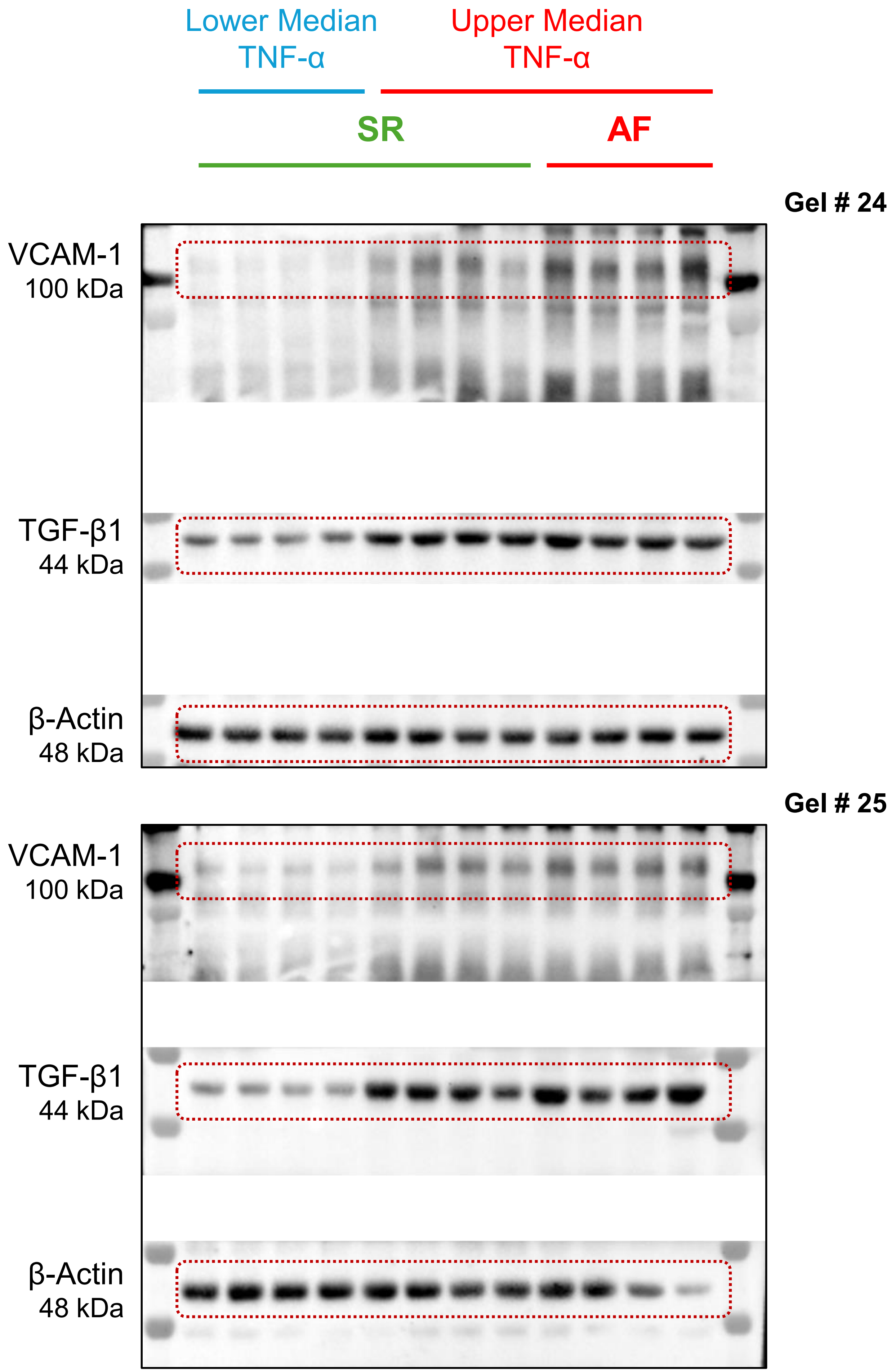
Lower Median
TNF-α
Upper Median
TNF-α
SR
AF
Gel # 24
VCAM-1
100 kDa
TGF-β1
44 kDa
β-Actin
48 kDa
Gel # 25
VCAM-1
100 kDa
TGF-β1
44 kDa
β-Actin
48 kDa

Figure S1A

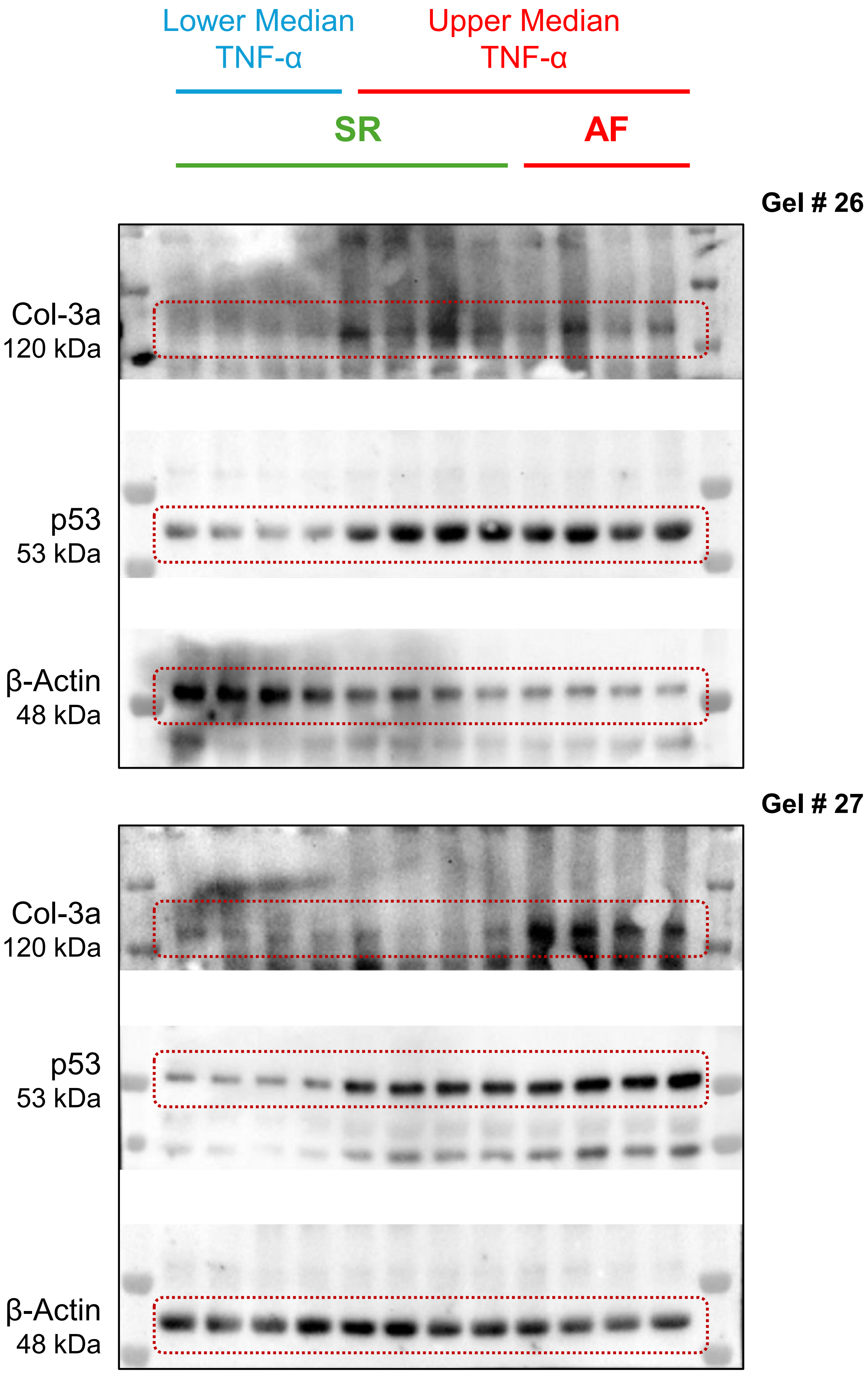
Lower Median
TNF-α
Upper Median
TNF-α
SR
AF
Gel # 26
Col-3a
120 kDa
p53
53 kDa
β-Actin
48 kDa
Gel # 27
Col-3a
120 kDa
p53
53 kDa
β-Actin
48 kDa

Figure S1A

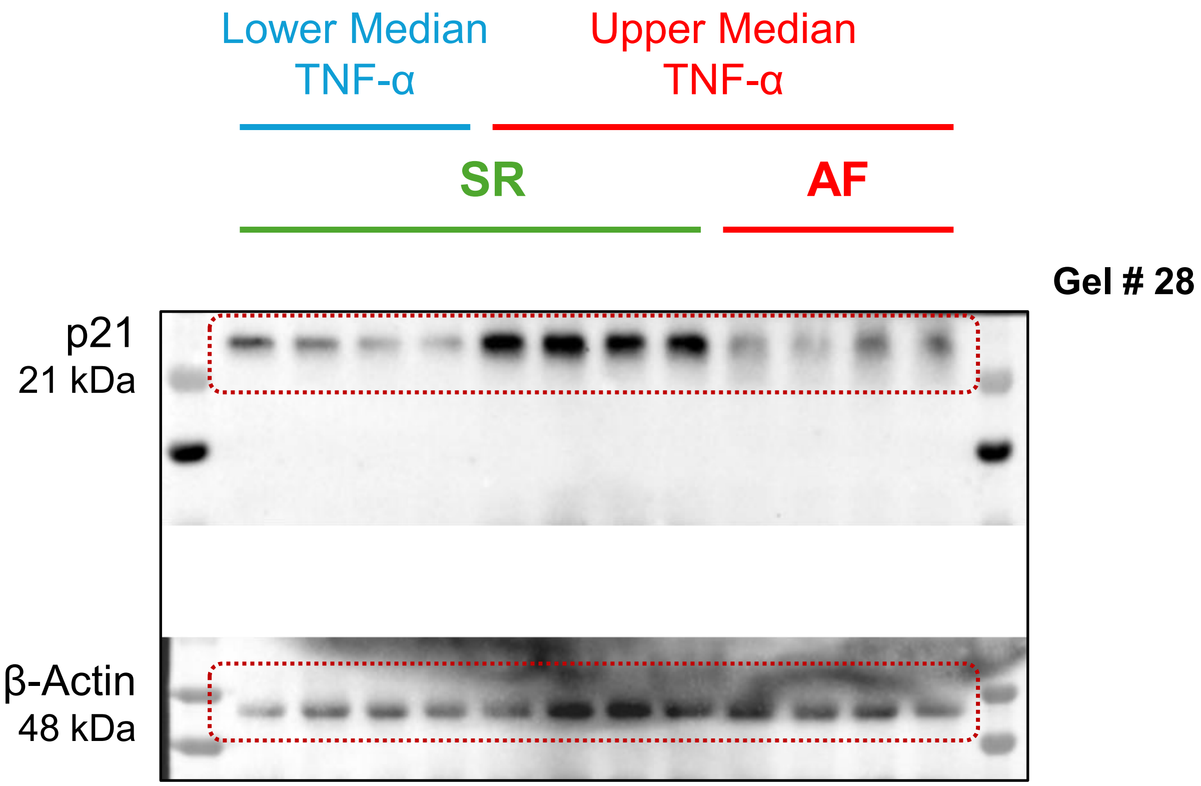

Lower Median
TNF-α
Upper Median
TNF-α
SR
AF
Gel # 28
p21
21 kDa
β-Actin
48 kDa

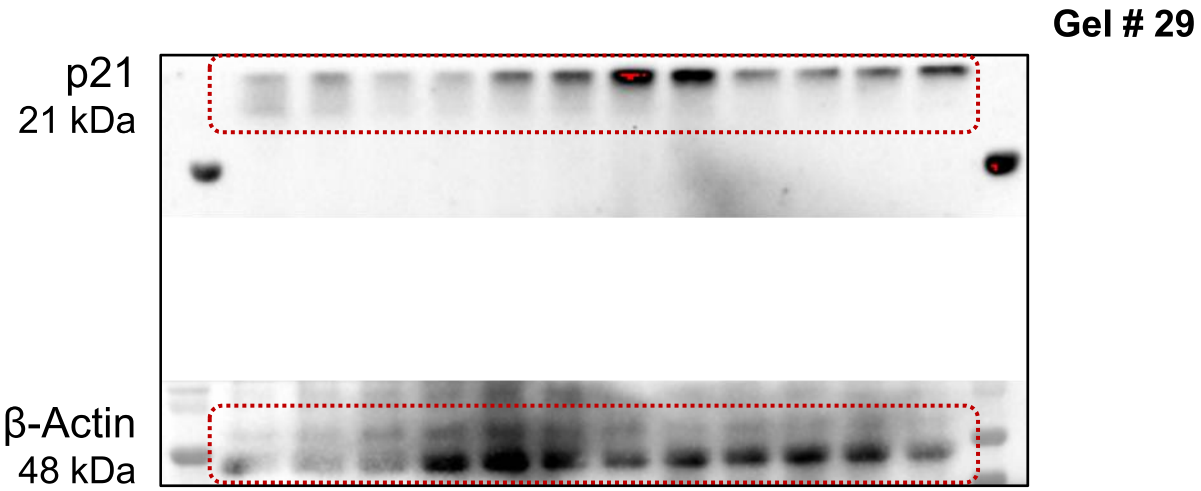

Gel # 29
p21
21 kDa
β-Actin
48 kDa

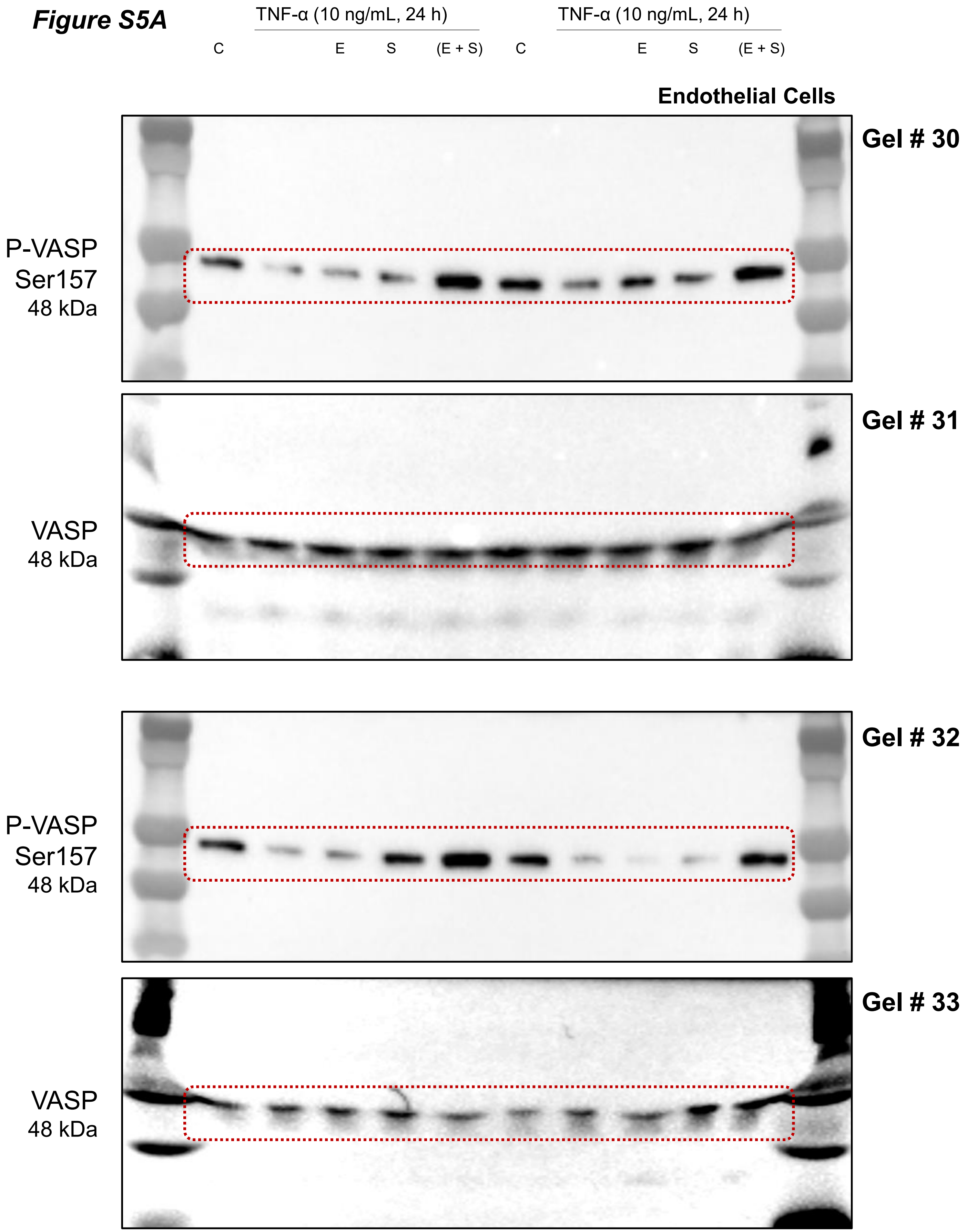
Figure S5A
TNF-α (10 ng/mL, 24 h)
TNF-α (10 ng/mL, 24 h)
C
E
S
(E + S)
C
E
S
(E + S)
Endothelial Cells
Gel # 30
P-VASP Ser157 48 kDa
Gel # 31
VASP 48 kDa
Gel # 32
P-VASP Ser157 48 kDa
Gel # 33
VASP 48 kDa

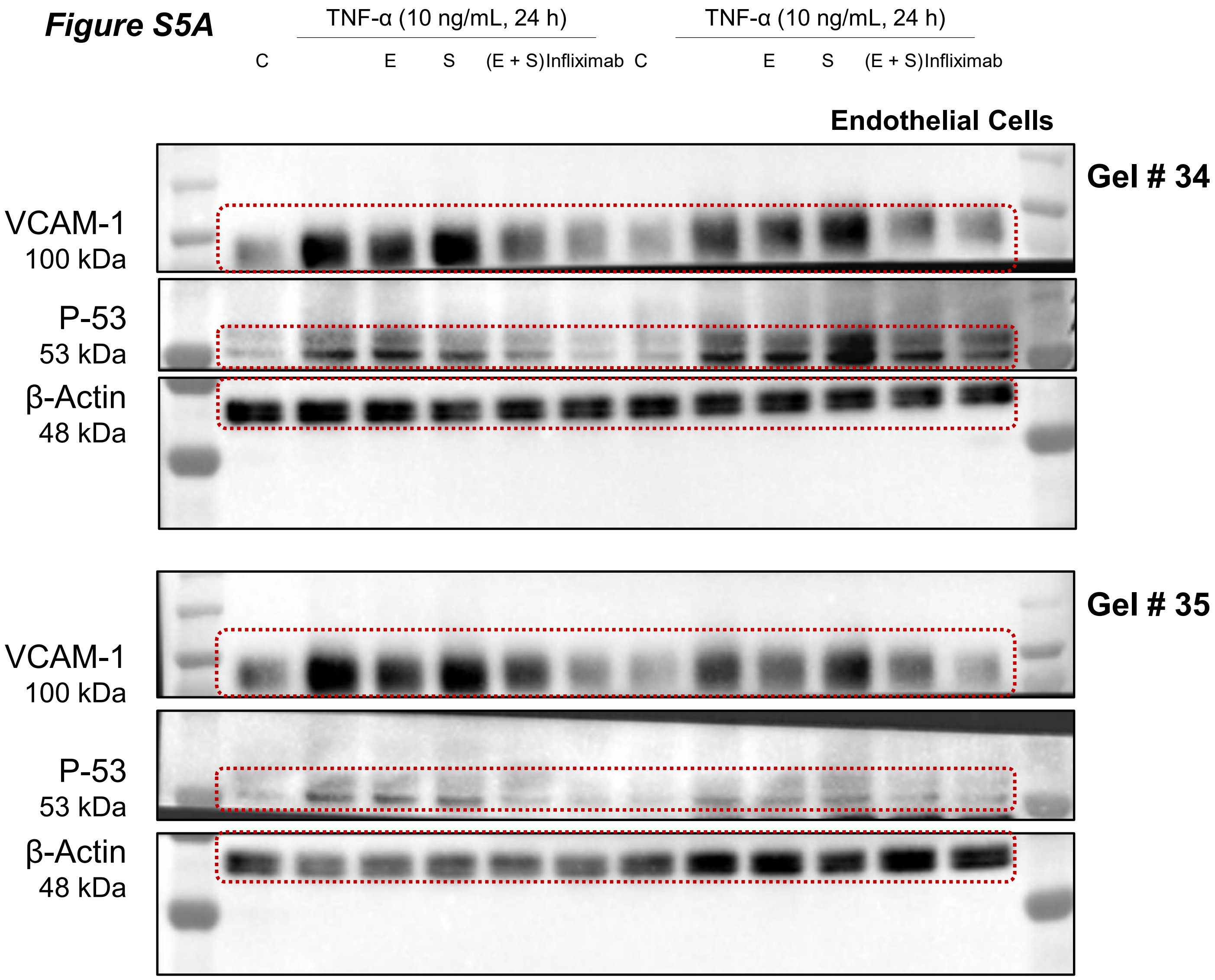
Figure S5A
TNF-α (10 ng/mL, 24 h)
TNF-α (10 ng/mL, 24 h)
C
E
S
(E + S)
Infliximab
C
E
S
(E + S)
Infliximab
Endothelial Cells
Gel # 34
VCAM-1
100 kDa
P-53
53 kDa
β-Actin
48 kDa
Gel # 35
VCAM-1
100 kDa
P-53
53 kDa
β-Actin
48 kDa

Figure S5B

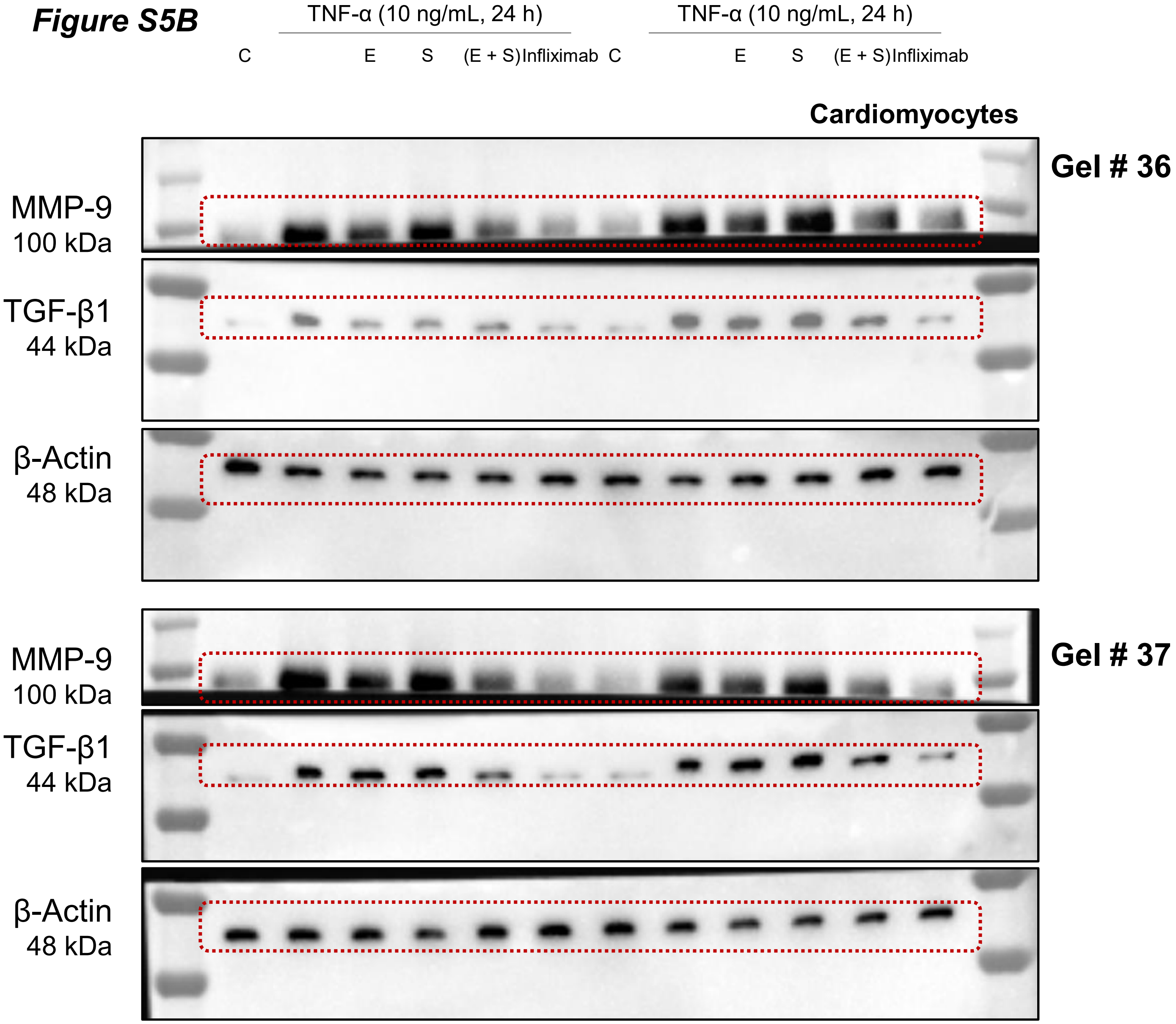
TNF-α (10 ng/mL, 24 h)
TNF-α (10 ng/mL, 24 h)
C
E
S
(E + S)
Infliximab
C
E
S
(E + S)
Infliximab
Cardiomyocytes
Gel # 36
MMP-9
100 kDa
TGF-β1
44 kDa
β-Actin
48 kDa
Gel # 37
MMP-9
100 kDa
TGF-β1
44 kDa
β-Actin
48 kDa

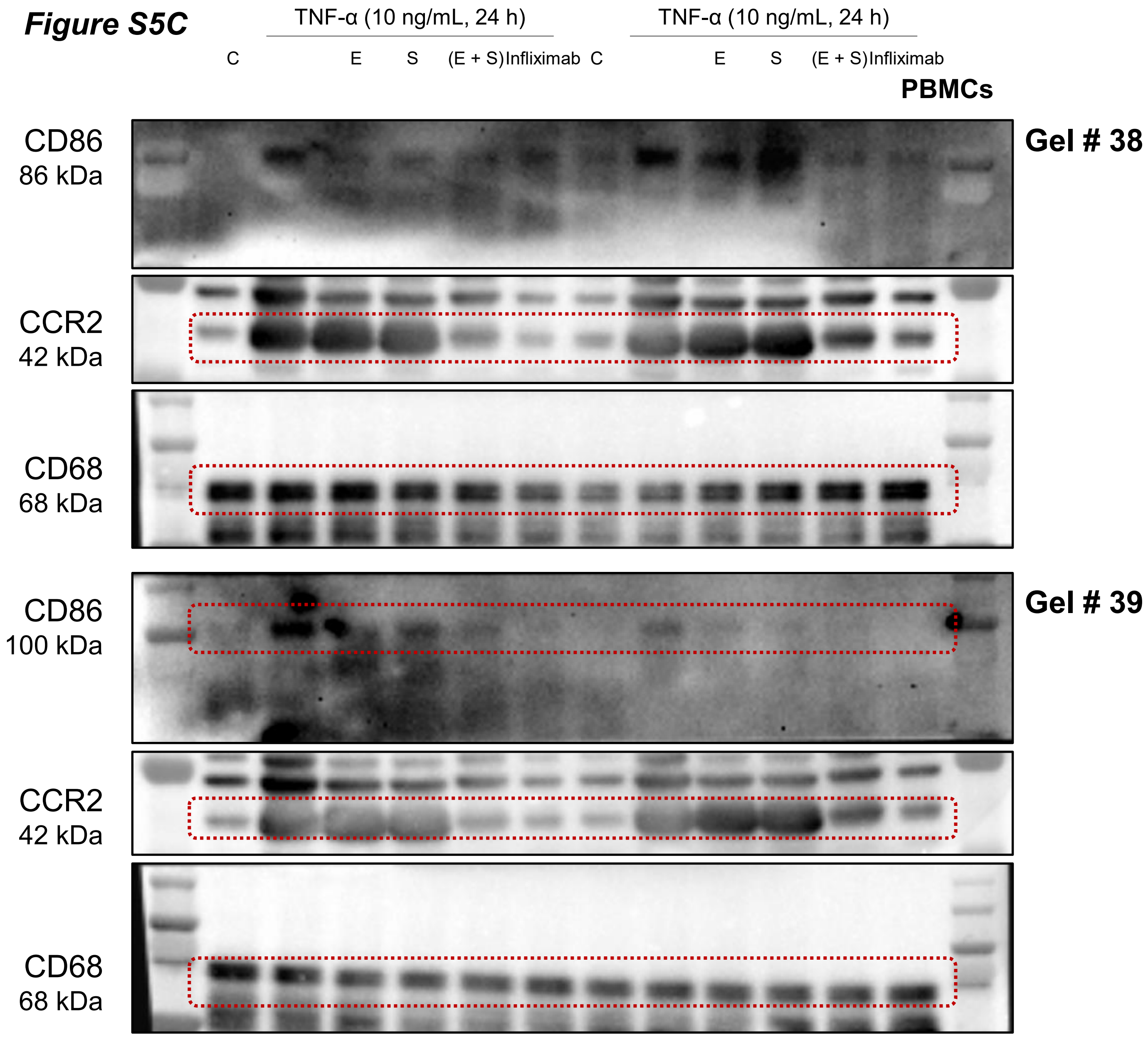
Figure S5C
TNF-α (10 ng/mL, 24 h)
TNF-α (10 ng/mL, 24 h)
C
E
S
(E + S)
Infliximab
C
E
S
(E + S)
Infliximab
PBMCs
CD86
86 kDa
Gel # 38
CCR2
42 kDa
CD68
68 kDa
CD86
100 kDa
Gel # 39
CCR2
42 kDa
CD68
68 kDa
